# Systematic Review: How the Attention-Deficit/Hyperactivity Disorder Polygenic Risk Score Adds to Our Understanding of ADHD and Associated Traits

**DOI:** 10.1016/j.jaac.2021.01.019

**Published:** 2021-02-04

**Authors:** Angelica Ronald, Nora de Bode, Tinca J.C. Polderman

**Affiliations:** University of London, United Kingdom.; Vrije Universiteit Amsterdam, the Netherlands, and Amsterdam UMC, the Netherlands.; Vrije Universiteit Amsterdam, the Netherlands, and Amsterdam UMC, the Netherlands.

**Keywords:** attention-deficit/hyperactivity disorder, genetics, neurodevelopment, comorbidity, psychiatry

## Abstract

**Objective:**

To investigate, by systematically reviewing the literature, whether the attention-deficit/hyperactivity disorder (ADHD) polygenic risk score (PRS) associates with ADHD and related traits in independent clinical and population samples.

**Method:**

PubMed, Embase and PsychoInfo were systematically searched, alongside study bibliographies. Quality assessments were conducted, and a best-evidence synthesis was applied. Studies were excluded when the predictor was not based on the latest ADHD genome-wide association study, when PRS was not based on genome-wide results, or when the study was a review. Initially, 197 studies were retrieved (February 22, 2020), and a second search (June 3, 2020) yielded a further 49 studies. From both searches, 57 studies were eligible, and 44 studies met inclusion criteria.

**Results:**

Included studies were published in the last 3 years. Over 80% of the studies were rated excellent, based on a standardized quality assessment. Evidence of associations between ADHD PRS and the following categories was strong: ADHD, ADHD traits, brain structure, education, externalizing behaviors, neuropsychological constructs, physical health, and socioeconomic status. Evidence for associations with addiction, autism, and mental health were mixed and were, so far, inconclusive. Odds ratios for PRS associating with ADHD ranged from 1.22% to 1.76%; variance explained in dimensional assessments of ADHD traits was 0.7% to 3.3%.

**Conclusion:**

A new wave of high-quality research using the ADHD PRS has emerged. Eventually, symptoms may be partly identified based on PRS, but the current ADHD PRS is useful for research purposes only. This review shows that the ADHD PRS is robust and reliable, associating not only with ADHD but many outcomes and challenges known to be linked to ADHD.

Attention-deficit/hyperactivity disorder (ADHD) is a neurodevelopmental disorder that affects approximately 5% of children and 2.5% of adults.^1^ Decades of past research have established the significant twin heritability of ADHD and family studies demonstrate its high familiality.^2,3^ More recently, significant single nucleotide polymorphism (SNP) heritability estimates for ADHD have been reported.^4^ Together this evidence supports the hypothesis that common genetic variants acting additively play a role in the causes of ADHD.^3^ In addition, twin, family, and molecular genetic studies suggest that these common variants may, to some degree, be shared with other conditions and traits, including autism and autistic traits,^5–10^ tobacco and alcohol use,^11,12^ and depressive and hypomanic symptoms.^13–15^

A genome-wide association study (GWAS) is the principal tool for identifying common genetic variants across the genome that influence complex traits.^16^ Following previous GWAS using comparatively smaller samples, the latest GWAS on individuals with ADHD (n = 20,183) and controls (n = 35,191) identified 12 independent loci associated with ADHD.^17^ Several characteristics of the study suggested that these findings were robust: for example, significant SNP heritability of 22% was reported, the genome-wide significant loci were replicated, and no marker demonstrated heterogeneity between studies.

GWAS data can be used to create a polygenic score, or, as often referred to in studies of psychopathological traits, a polygenic risk score (PRS). A PRS can estimate an individual’s genetic liability for a particular disorder or trait, based on current knowledge of the trait’s genetic architecture. Technically, a PRS is calculated as the weighted sum of the risk alleles carried by an individual which are associated with a disorder based on a GWAS. Demontis *et al.*^17^ reported that the variance in ADHD explained by their ADHD PRS was 5.5% in individuals of European ancestry (note that individuals of European ancestry were also used to calculate the score). In their samples, the PRS had an odds ratio (OR) of 1.56 between cases and controls and acted in a dose-dependent fashion: the higher the PRS, the higher the OR for having ADHD. A PRS can be calculated in any genotyped sample, and thus the degree to which the ADHD PRS associates both with ADHD as well as other phenotypes can be explored. The latter is interesting, given the reported co-occurrence and genetic overlap of ADHD with many other conditions or traits such as autism and substance use, as described above.

A PRS is thus a major methodological development, not only for the genetic field but also in terms of potential utility in a range of other research fields, because a PRS can be easily calculated in any genotyped sample. The potential of PRS for clinical utility, screening, and personalized health is currently a major topic of debate.^18,19^

Here we present a systematic review of all studies using the ADHD PRS based on the largest ADHD GWAS to date,^17^ and provide a systematic quality assessment of all included studies. In our review, we structured our results by the following outcome domains: diagnosed ADHD and ADHD traits (dimensional assessments of ADHD symptoms or traits), addiction, autism and autistic traits, brain-based (imaging) measures, educational attainment, externalizing behaviors, mental health, neuropsychological constructs, physical health, socioeconomic variables, and other (uncategorized) outcomes. Table S1, available online, provides a complete list of outcomes per category.

## METHOD

Details of the outline of our review and methods applied were preregistered with PROSPERO Framework (https://www.crd.york.ac.uk/PROSPERO) with registration number CRD42020176391 on April 28, 2020, and followed as registered, except for the following: (1) the study by Hayden *et al.* (2013), on which we based our quality assessments, proposes 6 quality domains; however, given some overlap in items of domains 1 and 2, we combined these, and thus used 5 domains instead of 6; and (2) given the sheer number of included studies resulting from the latest GWAS (N = 44) and the importance of an adequately powered GWAS to use the PRS reliably, we decided to exclude a systematic overview of studies based on older GWAS.

### Study Selection

PubMed, Embase, and PsychInfo were systematically searched for published, peer-reviewed studies written in English using the search terms: (“ADHD”[Title/Abstract] OR “Attention Deficit”[Title/Abstract] OR “Attention-Deficit”[Title/Abstract] OR “Hyperactivity”[Title/Abstract] OR “Hyperactive”[Title/Abstract] OR “attention deficit hyperactivity disorder“[Title/Abstract] OR “Attention problems”[Title/Abstract]) AND (“Polygenic risk score”[Title/Abstract] OR “Polygenic score”[Title/Abstract]). Bibliographies of selected studies were also searched (by NB). A first search was conducted February 22, 2020, and a second search June 3, 2020. All abstracts were inspected by 2 reviewers (TJCP and NB). Studies were excluded when the following conditions were met: (1) the predictor was not an ADHD PRS; (2) the PRS was not based on genome-wide results (but, for example, on a certain selection of SNPs); (3) the ADHD PRS was not based on the latest GWAS results of ADHD^17^; or (d) the study was a review.

**FIGURE 1:**
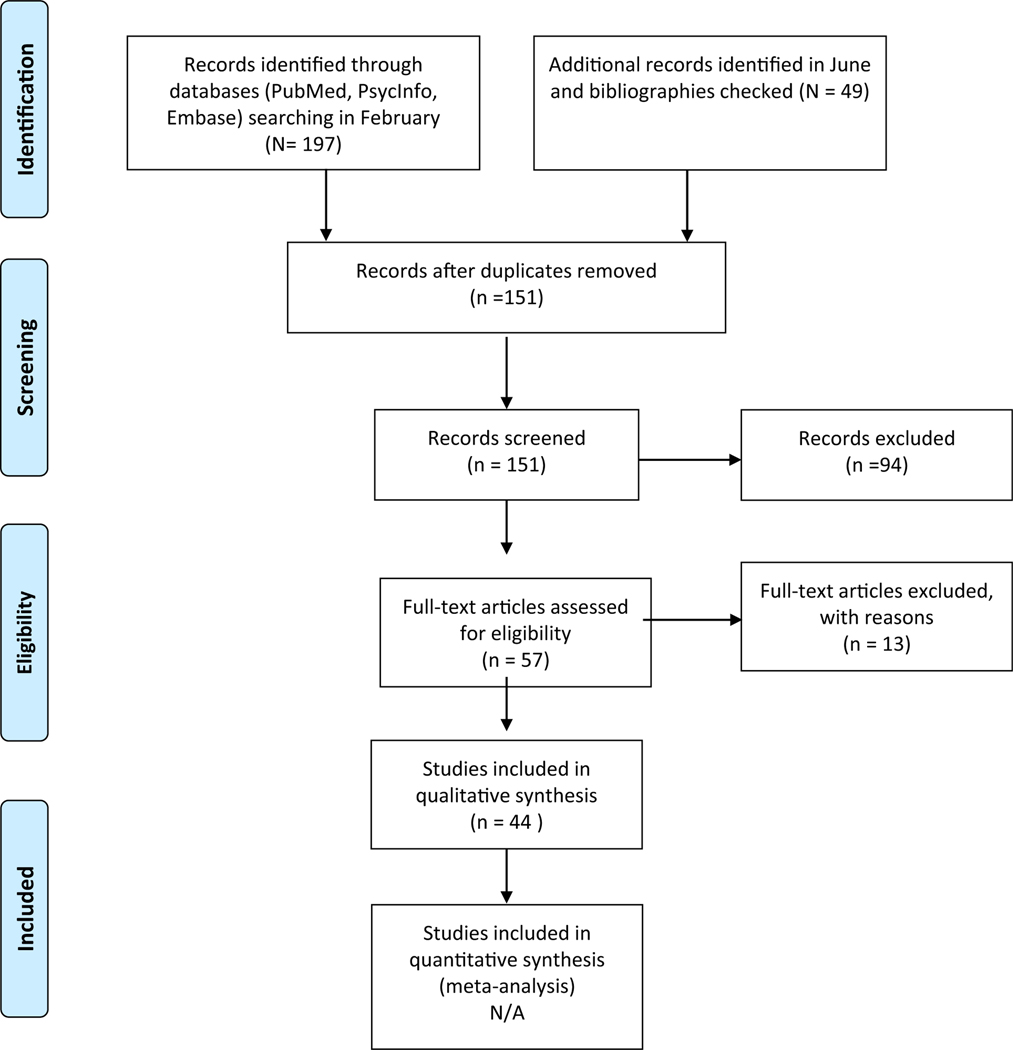
PRISMA Flow Diagram of Study Selection

Figure 1 provides a flow chart on the selection and reasons for exclusion of studies.

### The ADHD PRS

GWAS results allow the calculation of an individual PRS, which is based on the aggregate effect of common genetic variants that are associated with the trait of interest.^20,21^ The PRS can be used to test the association between the aggregated common genetic risk for ADHD and other human traits.

### Categorization of Outcome Measures

Categorization of outcomes was loosely based on *International Statistical Classification of Diseases* (ICD)/International Classification of Functioning, Disability and Health,^22,23^ but not completely for the following reasons. First, these classification systems would have meant losing specificity. Second, these systems are not designed specifically with ADHD in mind. For example, we chose to categorize externalizing behaviors and addiction as 2 specific categories because of their relevance to ADHD, rather than putting them under the umbrella category of mental health. Thus, some categories were made more or less specific, based on deliberation and consensus between authors AR and TJCP. Outcomes that were studied only once and did not fall readily into categories with other outcomes were placed in an “Other” category. Table S1, available online, provides an overview of outcome measures in each category.

### Quality Assessment

In general, scientific studies may encounter various biases resulting in potentially reduced validity and generalization of findings. Based on 2 studies by Hayden *et al.*,^24,25^ we set up a series of quality assessment criteria, clustered in 5 domains (listed below), to evaluate the quality of studies that we included in the current review.

1. *Study Participation.* A clear description of characteristics of the sample under study is key to evaluate how adequately the sample represents the population of interest, and how potential attrition may lead to selection bias affecting a proper representation.
2. *The ADHD PRS.* The validity and statistical power of a PRS depends on 2 crucial conditions. The first one is a powerful GWAS discovery sample, and the second one is proper quality control (QC) of the genetic data of the target sample under study. With the publication of the summary statistics of the largest GWAS on ADHD,^17^ for the first time, a reasonably powerful ADHD PRS became possible. Standard QC protocols are available^26^ to ensure that genetic data are correctly processed, and that important data checks are applied. Furthermore, when analyzing PRS data, a proper correction for population stratification should be applied.
3. *Assessment of Outcome Measures.* The current review includes multiple outcome measures that were tested for an association with the ADHD PRS. In the quality assessment, the validity and reliability of these outcome measures, either tested in the study or as citation to earlier publication, were the focus of evaluation.
4. *Confounding Factors.* Several confounding factors can play a role in the relation between the genetic risk for ADHD and the outcome measures. Given the variety of outcome measures, the focus of evaluation was on the following generic confounders: sex, age, socioeconomic status (SES), use of medication, and co-occurring disorders.
5. *Analysis and Data Presentation.* For a reader to judge the quality of a study, an adequate presentation of the statistical analyses and results is required. Of importance is also the target sample size, as sufficient statistical power is required to provide accurate conclusions on the relation between the ADHD PRS and outcome measures. Finally, multiple testing correction should be applied when more than 1 outcome measure is tested for an association with the predictor variable (ie, ADHD PRS).

A checklist consisting of criteria as described above was used to evaluate the quality of the 44 selected studies. Every item was rated positive (+), negative (−), or −/+ (ie, fulfilling part of the criteria) by 2 independent reviewers (TJCP and NB). In case of any disagreement between the reviewers, consensus was achieved by discussion. Studies were then ranked based on the number of biases. A bias was present when more than 50% of the criteria of 1 domain had a negative score. The highest quality was attained if at least 50% of the items of each domain were rated as being positive.^24,25^ Of note, because item M (treatment and comorbidity) could be rated only for the clinical samples and was not applicable (NA) for the population samples, this item was excluded from the bias count.

#### Best-Evidence Synthesis.

Within each of the categories, considerable variation was present in outcome measures. Therefore, we performed a best-evidence synthesis, to define the evidence for a true association between the ADHD PRS and each respective outcome category. The evidence for each category was determined by taking into account the number of studies evaluating this association, the quality of these studies, and the consistency of findings across studies.^27^ Based on this evaluation, 4 increasing levels of evidence were defined.^28^

## RESULTS

The 44 studies^29–71^ that met our inclusion criteria are listed in Table 1,^72–113,124–131^ and the results are summarized in Figure S1, available online. Categories of outcome(s) are given in the first column for each study. Samples are described in terms of name (where available), type, nationality, size, sex and age ranges. Choice of SNP *p* value threshold (pT) is listed in the fourth column. Outcomes, along with covariates, are listed in the fifth column. Results (sixth column) focus on the statistics, effect sizes, and their direction, for direct effects. The Results column describes any mediation analyses in terms of percent reduction in direct effect and outlines any sensitivity/replication analyses. Negative findings are reported, but statistics for negative findings are omitted for space considerations. The Results column also specifies the authors’ choice of significance threshold for testing the association between the ADHD PRS and outcome measure(s).

### Descriptives of Outcome Measures and Samples

Outcome measures were categorized in the following domains (number of studies shown in parentheses): diagnosed ADHD (n = 10), ADHD traits (n = 16); substance and non-substance-based addiction phenotypes (n = 8), autism spectrum disorders or autistic traits (n = 5), brain-based (imaging) variables (n = 8), educational attainment (n = 9), externalizing behaviors (n = 8), mental health (n = 11), neuropsychological constructs (n = 6), physical health (n = 4), socioeconomic variables (n = 4) and “other” (non-categorized outcomes) (n = 9).

Across the 44 studies, a total of 48 samples were used. Four studies included 2 samples, and it should be noted that these 48 samples are not all independent (see below). In terms of sample characteristics, 25 of the 48 samples (52%) were population samples, 16 (33%) were clinical samples, and 7 (15%) were community samples enriched for individuals with ADHD or mental illness. Children and adolescents (<18 years of age) made up just over half the samples (n = 25, 52%); 13 (27%) were adult samples; and the remaining (n = 10, 21%) included both children and adults. It was most common for samples to come from Europe (n = 25, 52%) followed by North America (n = 17, 35%), a mix of continents (n = 4, 9%), or Asia (n = 1, 2%), and 1 was missing the country of origin (2%). The samples used in more than one study were Avon Longitudinal Study of Parents and Children (ALSPAC) (6 studies), IMAGEN (3 studies), National Longitudinal Study of Adolescent to Adult Health (3 studies), Child and Adolescent Twin Study in Sweden (3 studies), Generation R (2 studies), community-based sample recruited close to Oregon Health and Science University USA (2 studies), and iPSYCH (2 studies).

#### Diagnosed ADHD.

The ADHD PRS consistently associated with diagnosed ADHD in all 10 studies. The odds ratios ranged from 1.22 to 1.76. This range omits one study that associated with ADHD within a cohort with bipolar disorder,^46^ and 2 studies that did not provide enough information to calculate odds ratios.^58,67^ Several studies^17,32^ showed, using deciles or groups based on low/medium/high scorers, that the ADHD PRS operated in a dose-dependent manner in terms of its influence on ADHD status.

In terms of ADHD and co-occurring conditions, ADHD PRS was associated with having combined ADHD and ASD in a multiplex family design including unaffected relatives and relatives with either or both conditions.^68^ The ADHD PRS did not differentiate bipolar disorder cases with ADHD from bipolar disorder cases without ADHD.^2^ In the context of other psychiatric disorders, ADHD PRS was associated with ADHD when controls were individuals with other psychiatric disorders.^69^

#### ADHD Traits.

This was the most commonly studied outcome, and all studies found positive significant associations with the ADHD PRS (16 studies). Percent variance explained in ADHD traits by the ADHD PRS ranged from 0.7% to 3.3%. These values were either directly reported or were converted from correlations provided in the studies. Five studies that reported on ADHD traits^46,36,46,63,66,120^ were omitted from this range because their study designs were different (eg, they investigated only subscales, they investigated familial effects, the sample was bipolar disorder cases).

Four of these studies investigated the ADHD trait subscales separately, namely hyperactivity/impulsivity and inattention. Two studies (50%) found that the ADHD PRS was positively associated with higher scores on both subscales,^33,53^ whereas 2 (50%) found that the ADHD PRS was positively associated with the hyperactivity/impulsivity subscale but not significantly associated with inattention.^43,63^

#### Addiction.

A range of addiction phenotypes were studied: 7 studies on substance-related addiction^32,40,47, 52,55,58^ and one study on a non-substance–related addiction (ie, gambling).^36^ Three studies did not find the ADHD PRS associated with their addiction phenotypes (which focused on gambling behaviors, substance abuse, and marijuana use disorders). The other 5 studies reported all or some significant positive associations, including with cocaine dependence, substance use disorders, alcohol (intake frequency and alcohol-related diagnoses), smoking, cannabis use disorder, use of illicit drugs, and severity of addiction.

#### Autism Spectrum Disorders and Autistic Traits.

Five studies investigated diagnosed autism or autistic traits. Only one study (on autism) reported a significant positive association with the ADHD PRS, although full effect sizes were not provided.^67^ One study on autistic traits reported a significant positive association in male participants only but the effect was not present for the full sample or in female participants.^64^

#### Brain-Based (Imaging) Phenotypes.

All but 1 of the 8 studies on brain structure or connectivity^29,49,54,56,63,65,66^ reported significant associations with the ADHD PRS. Five of these also conducted mediation analyses, within which there was a variety of evidence that brain structure mediates the association between the ADHD PRS and ADHD. The specific brain-based outcomes are listed in Table S1, available online: 7 of the 8 studies included structural measurements, including both gross indices such as gray matter volume or more detailed measurements such as subcortical structures; 2 studies included functional parameters.

#### Educational Attainment.

Seven of the 9 studies analysing educational attainment reported that the ADHD PRS was associated with lower educational attainment.^32,34,39,43,60,63,68^ One nonsignificant finding came from a study that did not test a straightforward association but separated the PRS into transmitted and nontransmitted alleles^50^ and thus tested 2 separate PRSs for their association with educational attainment, which reduces power.

#### Externalizing Behaviors.

The ADHD PRS was significantly positively associated with a range of externalizing behaviors across 8 studies (cross-sectional assessments of irritability, surgency, impulsivity, aggression, and risk taking), and there was evidence that the ADHD PRS was also associated with trajectories of increasing and persistent irritability and with high decreasing trajectories of externalizing behaviors.^38,43–45,47,49,68,71^

#### Mental Health.

Within this category, there were 11 studies^32,39,41,43,46,48,51,60,68–70^ with a broad range of phenotypes but not consistent significant findings. The ADHD PRS was significantly positively associated with the general psychopathology factor in children (also referred to as the *p* factor).^48^ Higher ADHD PRS was associated with a bipolar disorder subtype combined with ADHD when compared to unaffected controls but did not associate with bipolar disorder when compared to unaffected controls. Four studies explored schizophrenia or subthreshold psychotic experiences, and none reported a significant association with the ADHD PRS. In terms of anxiety, depression, and neuroticism, results were mixed. For example, the ADHD PRS was associated with higher neuroticism in one study of older adults,^58^ and with more perceived stress in another study,^32^ but was not associated with neuroticism in a youth sample.^51^ The ADHD PRS positively associated with depression in a study of older adults.^58^ In a study of children, the ADHD PRS was positively associated with any anxiety or depressive disorder, but there were some nonsignificant associations for specific disorders dependent on the type of diagnostic tool that was used.^69^ In terms of trajectories of depression across ages 10 to 18 years in youths, the higher scores on the ADHD PRS associated with an early-adolescence–onset depression class but not late-onset depression.^70^ The ADHD PRS also positively associated with a range of eating disorder traits in youth.^51^

#### Neuropsychological Constructs.

Of the 6 studies on neuropsychological constructs,^37,43,57,63,65,66^ 5 studies included working memory and all reported significant associations between poorer working memory and higher ADHD PRS. Other neuropsychological constructs studied in relation to the ADHD PRS were executive function outcomes (all nonsignificant); vigilance/arousal (significant negative association); output speed, mental clock, and response inhibition (all nonsignificant); focused attention and delay discounting (significant). Three studies used neuropsychological variables such as working memory as mediators in models of the association between the ADHD PRS and ADHD^57,63,66^ (Table 1).

#### Physical Health.

Of the 4 studies exploring physical health,^32,49,60,68^ 3 studies included body mass index (BMI), and all showed a significant positive association with ADHD PRS (albeit using different methods) (Table 1). The other physical health phenotypes studied were height^68^ (mixed evidence), hypertension, and blood cholesterol^32^ (no associations for either in PRS group comparisons).

#### Socioeconomic Status.

Four studies^35,41,58,66^ tested whether the PRS associated with variables related to socioeconomic status. All studies showed a significant association with the ADHD PRS being negatively associated with socioeconomic status (SES). The study by Selzam *et al.*^60^ showed a significant negative association with SES in both the between and within family design.

#### Other (Noncategorized) Outcomes.

In terms of the 9 noncategorized outcomes,^30,42,46,47,52,59,60,61,71^ the ADHD PRS was positively associated with being bullied,^61^ bullying chronicity,^61^ and a victimization adversity scale,^71^ a total adversity scale,^71^ earlier age of onset of bipolar disorder,^46^ reduced participation in research studies,^59^ selected methylation probes,^62^ reduced parental monitoring,^52^ and risk of parental mental disorder or substance use disorder.^47^ The ADHD PRS did not associate with infant neuromotor functioning,^64^ or community disadvantage, and did not associate with ADHD traits in youths with mild traumatic brain injury.^30^

### Quality Assessments

Table 2 shows the items of the quality assessment (QA), and Table 3 shows the levels of evidence. The results of the QA for each study are presented in Table 4. Three studies had 2 biases, and 5 studies had 1 bias, leaving 36 studies without any notable bias. Studies that did have 1 or 2 biases were randomly distributed across categories. Item K (correction for age, sex, and socioeconomic status) was rated most often as −/+, because the majority of studies did not correct for socioeconomic status and this criterion was not relevant for the SES outcome category. Furthermore, sample sizes of target samples were, in some studies, n < 500, which we considered small, although expected effect sizes may differ between outcome measures.

The criteria from the best-evidence synthesis (Table 3) suggested that the evidence for an association between the ADHD PRS and the following outcome categories was “strong”: diagnosed ADHD, ADHD traits, brain-based imaging phenotypes, education, externalizing behaviors, neuropsychological constructs, physical health, and socio-economic status. The evidence was “inconclusive” for the addiction, autism and autistic traits, and mental health categories. The “other” category was not included in the best-evidence synthesis.

## DISCUSSION

Overall, our literature review demonstrates that the ADHD polygenic risk score (PRS) is reliable, robust, and operates in a dose-dependent manner. We found strong evidence from our best-evidence synthesis that the common genetic variants underlying ADHD, as captured by the ADHD PRS, associated not only with diagnosed ADHD but also with more dimensional ADHD traits, more externalizing behaviors, impaired working memory and education attainment, reduced brain volume, higher body mass index, and reduced socioeconomic status. These findings illustrate that the well-known phenotypic associations between ADHD and many of these phenotypes, stemming from decades of research in epidemiology and developmental psychology, may partly be explained by shared genetic effects. There is an emerging literature, albeit not with conclusive evidence according to our best-evidence synthesis, suggesting that outcomes beyond childhood, such as addiction and adult mental health, may also associate with the ADHD PRS. Some phenotypic outcomes are less well researched than others; this led to quite broad outcome categories in some instances (eg, physical health), whereas others were able to be more specific because of the larger body of literature (diagnosed ADHD, ADHD traits, externalizing behaviors, and addiction).

The ADHD PRS appears to carry a degree of specificity both in relation to other PRS, in terms of the wider context of neurodevelopment and mental health, and in its capacity to significantly associate with only ADHD-relevant phenotypes. Illustrating this, some studies used a multi-PRS model and found that the signal from the ADHD PRS remained significant when controlling for other PRSs.^48,61,70^ In the wider context of neurodevelopment and mental health, the ADHD PRS often did not associate with other conditions such as autism and schizophrenia^31,41,67,68^ or family history for mental health conditions,^70,71^ and it associated with bipolar disorder only when it co-occurred with ADHD.^46^ When studies included negative control traits, they invariably did not, as predicted, associate with the ADHD PRS.^43,68^ Yet, there were also some surprising and novel cross-disorder findings: for example, the ADHD PRS was associated with eating disorder traits in adolescents.^51^ However, it should be noted that the effect sizes of these eating disorder trait associations (0.10%–0.13%) were at least 5 times lower than the lowest estimated effect size for ADHD PRS associating with ADHD traits (the range being 0.7%–3.3%). Thus, the literature supports the validity of the ADHD PRS: the most consistent and strongest associations were with diagnosed ADHD and ADHD traits.

As a literature, the use of the ADHD PRS is fast growing (44 studies in less than 3 years), of high quality (as indicated by our QA assessment), with both breadth (in terms of the wide range of outcome phenotypes) and depth (in terms of both replication within and between studies and extensive analytic protocols). The risk of false-positive results in PRS studies is potentially high from a combination of authors being free to choose multiple significance thresholds on which to test associations and multiple phenotypes. Most studies appeared to have clear measures in place to avoid false-positive results: as noted in Table 1, the majority of studies used some form of significance criterion correction and stated their SNP-based significance thresholds (pT); most selected a single pT and provided a justification for their choice; and many included sensitivity analyses to ensure that results were robust. Common sensitivity analyses included repeating analyses on other pT, on different ancestral groups within the sample, excluding children on medication and in community samples by excluding children with diagnosed ADHD.

Within the studies on non-ADHD disorders, the ADHD PRS appears to be useful for predicting trajectories. Specifically, the ADHD PRS appears to have transdiagnostic utility in characterizing subgroups of individuals with early-onset symptoms in non-ADHD conditions. For example, although ADHD PRS did not associate with schizophrenia, within a schizophrenia sample, it associated with cognitive trajectory from adolescence into adulthood, being most strongly associated with the subgroup with (earliest) preadolescent cognitive impairment.^39^ The ADHD PRS did not associate with bipolar disorder, but it associated with an earlier age of onset within bipolar disorder cases.^46^ Finally, the ADHD PRS associated with an early-onset depression trajectory class but not a later-onset depression trajectory class in youths assessed longitudinally at ages 10 to 18 years.^70^

The ADHD PRS has been used in several studies to investigate gene—environment correlation, namely, genetic influences on environmental exposure. Direct effects of the ADHD PRS are reported on lower socioeconomic status,^60^ lower parental education and income,^47^ worse labor market outcomes,^35^ adversity,^71^ and bullying victimization.^61,71^ Two studies went beyond direct genetic effects by applying within-family analytic designs. De Zeeuw *et al.* split the ADHD PRS into transmitted and nontransmitted alleles to test for a process termed “genetic nurture.”^50,122^ They did not find that the parents’ nontransmitted ADHD PRS (ie, the part of the ADHD PRS inherited by parents but not transmitted to their offspring) influenced the offspring’s ADHD symptoms. The more elaborate design by Selzam *et al.* involved splitting up the covariance within their sample of twin siblings into between-family and within-family effects.^60^ The authors concluded that some of the association between the ADHD PRS and educational attainment might be due to passive geneLenvironment correlation effects. It is important to note, going forward, that part of the signal in a PRS may be correlated with socioeconomic factors.

The reviewed literature included multiple studies investigating PRS—brain—behavior pathways relevant to ADHD. This new literature is worth highlighting in part because most attempts pre-GWAS to link neuroimaging data simultaneously to both genetics and behavior could be considered noble failures, beset with issues of multiple testing and low power.^123,124^ The studies in our review demonstrate that reduced brain volume mediates the association between the ADHD PRS and ADHD. For example, in 1 recent study, the ADHD PRS was negatively associated with total brain volume, and total brain volume accounted for 16% of the association between ADHD PRS and ADHD diagnosis.^42^ Mediation was also used successfully in other categories. For example, in the neuropsychological category,^57^ the association between the ADHD PRS and ADHD diagnosis was mediated by working memory and arousal alertness latent variables. In the externalizing category, it was shown that externalizing symptoms mediated the association between the ADHD PRS and adversity.^71^

The ADHD PRS can teach us about the core aspects of ADHD and its nosology. Eventually, the ADHD PRS may contribute to the clinical picture for individual patients, but because of the current small effect sizes, the ADHD PRS is useful for research purposes only. Given the presence of the 3 presentations of ADHD in the *DSM-5* (combined, predominantly inattentive, predominantly hyperactive—impulsive), it is perhaps surprising that only 4 of the 16 studies on ADHD traits investigated associations of the ADHD PRS separately by ADHD symptom domain.^33,43,53,63^ Another study that touched on nosology proposed that emotional dysregulation should be considered a core component of ADHD, in light of their finding that an ADHD subgroup with emotional dysregulation had a higher ADHD PRS score compared to other ADHD subgroups.^38^

Given the variety of outcome categories and the variety of outcome measures within categories, a meta-analysis was not conducted. Still, we report the current range in effect sizes for ADHD and ADHD traits. Furthermore, to obtain insights into the reliability and strength of the associations, we applied a best-evidence synthesis that was based on a careful and systematic quality assessment of all studies. Other limitations of our systematic review include the fact that it is difficult to estimate the power of studies based on their target sample size without knowing the expected effect size of an association.^125^ We restricted our review to studies using PRS based on the largest and latest GWAS on diagnosed ADHD. This meant excluding studies on PRS derived from ADHD traits or ADHD traits combined with diagnosed ADHD,^126^ and studies using older ADHD PRS (eg, reviewed by Vuijk *et al.*^43^) as well as studies using a cross-disorder PRS that includes the ADHD PRS. Not all of the 44 studies are completely independent because of some partially or completely overlapping samples. For most categories, every study was based on a different sample. However, it should be noted that 3 of the 10 studies on mental health outcomes used the ALSPAC sample, and 2 studies used the Child and Adolescent Twin Study in Sweden (CATSS) sample. Nonetheless, given that the evidence for the mental health category was mixed and inconclusive, the repeated use of the ALSPAC and CATSS sample in this category does not appear to have inflated the consistency of the evidence for these categories. In terms of the other categories, 2 of the 16 ADHD trait studies and 3 of the 8 studies on brain-based outcomes used the IMAGEN sample, and 2 of the 8 addiction studies used the Add Health sample. Finally, we included studies based on clinical, enriched, and population-based samples. We found no differences between the samples in their associations with the outcome measures: in the outcome measures for which we observed inconclusive results (ie, autism, addiction, and mental health), significant associations did not cluster by sample type.

While emphasizing the high quality of most of the reviewed literature and the strong evidence that has emerged for associations of the ADHD PRS with outcomes, a number of limitations and suggestions for improvements in this field of research are noted. Ideally, field standard approaches in terms of the method of analyzing PRSs would be devised, and pre-registration is essential. At present, there are multiple approaches and methods that are only beginning to be formally compared.^127^ The selected pT and the justifications for selection of pT varied widely across studies: some selected *p* < .05 to avoid over-fitting, some selected the pT that most accurately predicted ADHD in the work by Demontis *et al.*,^17^ some u sed pT = 1 to capture all variance, and others applied ranges of multiple pT. When studies did not specify their selected *p* value threshold, we had to select one from which to report the results, and this may have exacerbated false-positive results. A reference-standardized approach may be needed to compare PRS across different target samples, to avoid factors often specific to the target sample influencing PRS, including the variants considered, linkage disequilibrium, and allele frequency estimates.^127^ It will be exciting to see future work that combines the ADHD PRS with rare variation and copy number variation or that incorporates the sex chromosomes.

As shown in Table 1, the majority of this literature was conducted in samples of European ancestry: of the 44 studies, 77% (n = 34 studies) had European ancestry, 91% (n = 41) had most or all European ancestry, one study had missing ancestry information, and 5% (n = 2) had participants with non-European ancestry (Japanese and African American, respectively). To maximize the value of the data, some studies ran sensitivity analyses on their samples based on different ancestral populations.^32,57^ Major initiatives in terms of both sample ascertainment and method development are needed to ensure that the genetic architecture of ADHD is understood regardless of the ancestry of the population under study.^128^ At present, the literature on the ADHD PRS offers only partial insight globally, because roughly only 1 in 20 studies on the current ADHD PRS to date uses participants with non-European ancestry.

It is noted that some of the associations identified here are largely supported by studies using LD score regression as well as from past twin studies. LD score regression provides an estimate of the degree of shared genetic effects in common genetic architecture. PRS studies are distinguishable for several reasons, including the fact that they allow tests for associations between ADHD and other phenotypes that currently lack a large GWAS. Furthermore, as seen in this review, PRS can also easily be manipulated within more complex analytic frameworks to test more complex hypotheses, such as analyses involving trajectory modeling or mediation models.

In terms of individual prediction, the existing literature only goes as far as to compare groups scoring high, medium, and low on the ADHD PRS in a small number of our reviewed studies. The ADHD PRS cannot yet accurately predict individual outcomes, and a PRS is only as accurate as the discovery sample from which it is computed. Anyone who has used direct-to-consumer testing can upload their genetic data on a new tool to calculate their own ADHD PRS.^129^ Most individuals who score high on the current ADHD PRS will not develop ADHD because the signal is too weak. There is a strong need for public engagement and public debate on the clinical usability of PRS.^130^ It is possible that a more predictive ADHD PRS will be used in the future, in combination with other known risk factors and clinical features, to support health services with prediction, diagnosis, and intervention.^131^ As pointed out elsewhere, there are some similarities between existing successful health screening practices (such as the newborn Apgar score and neonatal blood spot screening) with how a PRS would be obtained and could work in practice.^19^

In sum, our review identified 44 relevant studies and demonstrates the accumulation of strong evidence that the ADHD PRS associates not only with ADHD and ADHD traits, but also with reduced brain volume, lower education attainment, more externalizing behaviors, impaired working memory, higher body mass index, and lower socioeconomic status. Alongside these direct effects, the ADHD PRS is being used to reveal more complex processes such gene—environment correlations and that the ADHD PRS influences ADHD symptoms via effects on brain structure. Genetic associations that might have been expected based on past literature, such as between the ADHD PRS and addiction, autism, and mental health, are so far inconclusive from the available evidence. In the context of other known risk factors for ADHD, the ADHD PRS does not have the largest effect size. Nevertheless, the ADHD PRS brings advantages in terms of being based on genetic variants, and thus being biologically based, possessing a degree of causality and being unchanging across the lifespan (unlike most other risk factors). The estimated SNP heritability of ADHD is larger than the percent variance explained by the current ADHD PRS. We can expect, therefore, that with a larger GWAS of ADHD, a more accurate and predictive PRS will emerge going forward.

## Supplementary Material

Supp Mat 1

Supp Mat 2

## Figures and Tables

**TABLE 1:** Description of Included Studies on the Association Between the Attention-Deficit/Hyperactivity Disorder (ADHD) Polygenic Risk Score (PRS) and Outcomes Measures

Category	Study	Sample	ADHD PRS *p* threshold(s) (pT)	Outcome measures and covariates	Results
ADHDt, BRAIN	1. Albaugh *et al.* 2019^29^	IMAGEN Study, France, UK, Ireland, Germany N = 1471–1597 participants, age range = 12 – 16 y 52% female, 48% male Population sample Western European ancestry	PRS calculation based on pT = 0.05	MRI: neuroanatomic imaging, and imaging of white matter tract microstructure correlates of ADHD symptomatology ADHD traits: composite score of the Development and Well-Being Assessment (DAWBA)^72^ and the Strengths and Difficulties Questionnaire^73^ (SDQ) Covariates: age, sex, site, socioeconomic status, pubertal stage, total brain volume, PCs	ADHD PRS was significantly associated with ADHD traits in participants with available cortical thickness data (*r* = 0.125, *p* < .001), and with available diffusion data (*r* = 0.137, *p* < .001). ADHD PRS predicted neuroanatomic imaging, and imaging of white matter tract microstructure, as it significantly associated with the ADHD dimensional symptom score (b = −0.044, *p* = .045). Sex did not significantly moderate the association between PRS score and mean FA. Repeated analyses with the PRS SNP threshold changed to *p* < .01 and <0.10 showed consistent results, as did repeated analyses controlling for IQ. In voxelwise analysis within white matter skeleton regions, the neuroanatomic imaging, and imaging of white matter tract microstructure association was significantly associated with ADHD traits. Strongest associations (*p* < .001, uncorrected) were revealed in portions of the left inferior fronto-occipital, superior longitudinal, and inferior longitudinal fasciculi. ADHD PRS was not associated with cortical thickness in the cortical areas that were significantly associated with ADHD traits Statistical thresholds were *p* < .05 familywise error corrected, and brain data were threshold-free cluster enhancement corrected.
ADHDt, OTHER	2. Stojanovski *et al.* 2019^30^	Philadelphia Neurodevelopmental Cohort, USA N = 1,233 participants with no traumatic brain injury (TBI); N = 204 with mild TBI; 79 with high risk TBI. Age range = 8 – 21 y 47% female, 53% male Population sample European ancestry	PRS calculation based on pT = 1	Mild traumatic brain injury (TBI), and ADHD symptoms Structured interview assessed symptoms and criteria corresponding to ADHD diagnostic criteria ADHD (*DSM5–5*^74^) Covariates: age, sex, parental education, PCs	A significant interaction between ADHD PRS and group (mild TBI vs. no TBI) (t1427 = −2.1, *p* = .04). ADHD PRS showed a positive association with ADHD symptom score in youths without TBI (t1224 = 3.5, **Δ** R^2^ = 0.009%, *p* = .004) and no association with ADHD symptom score in those with mild TBI (t196 = 20.4, **Δ** R^2^ = 2.004%, *p* = .70). Sensitivity analyses were run excluding individuals with ADHD and individuals taking medication for emotions or behavior issues. Both of these analyses showed a similar interaction pattern, but the interaction did not reach significance. A *p* < .05 significance threshold was used because only 1 comparison was run.
ADHD, ASD	3. Jansen *et al.* 2019^31^	Inside Out Sample, The Netherlands Clinical sample N = 688, age range: 2–18 y (mean: 9.06, SD: 2.66) ADHD only sample: n = 280 participants, 25% female, 75% male; ASD only sample: n = 295 participants, 27% female, 73% male. Combined sample (ASD only and ADHD only samples above plus n = 113 participants with both ASD+ADHD), 24% female, 76% male All European ancestry Control sample from the Netherlands, N = 943, age range 17 – 79 y, 37% male, 63% female	PRS calculation based on 8 pT (0.01–1)	DSM-IV^75^ ADHD diagnosis, ASD diagnosis, and combined (ASD, ADHD, or both diagnoses) Parent-rated Child Behavior Check-List/6–18 (CBCL)^76^ Covariates: age, PCs	ADHD PRS predicted both the combined (ADHD and/or ASD) diagnoses (OR = 1.28; *p* = 1.3 x 10–3) and ADHD-only (OR = 1.4; *p* = 3.6 x 10 – 4), but not ASD- only. At the optimal p value threshold, R^2^ = 0.02% for the combined (ADHD and/or ASD) sample and R^2^ = 0.045% for the ADHD-only sample. Planned sensitivity analyses between ADHD symptom severity scales and PRS were not run because of low correlations. Significance threshold was p < .05 Bonferroni corrected for 72 tests.
ADHD, ADDICTION, EA, MH, PHYSICAL	4. Li 2019a^32^	National Longitudinal Study of Adolescent to Adult Health (Add Health), USA N = 7088 participants, mean age: 29.00 y (SD: 1.74) 54% female, 46% male Population sample 63.6% Caucasian (including Hispanic), 20.7% African American, 0.2% Native American, 5.1% Asian, and 10.3% "other"	PRS calculation based on pT = 1. PRS groups defined as low (<20th percentile), medium (21st–70th percentiles), and high (>80th percentile) compared on outcomes	ADHD diagnosis based on retrospectively self-reported ADHD symptoms keyed to the *DSM-IV*^75^ Lifetime *DSM-IV* criteria for alcohol abuse or dependence were assessed as the presence of at least 1 of the 4 items pertaining to alcohol abuse, and/or 3 of the 7 items pertaining to alcohol dependence occurring together in 12-mo period. Educational attainment, measured by the question "What is the highest level of education that you have achieved to date?" Scale ranged from 1 (8th grade or less) to 10 (some graduate training beyond a master's degree). Cognitive ability, measured by Add Health Picture Vocabulary Test (AH PVT)^77^ Mental health, measured by diagnoses based on the DSM- IV,^75^ the Center for Epidemiologic Studies Depression (CES-D) scale,^78^ and an abbreviated 4-item version of the Cohen's Perceived Stress Scale.^79^ Also, it was asked whether the participant was "ever arrested." Physical health determined based on body mass index (BMI) and patients reported whether they had hypertension or high blood cholesterol as reported by a doctor. Covariates: age, sex PCs	ADHD PRS was associated with ADHD diagnosis (OR = 1.22, *p* < 0.001). In terms of probability of ADHD by PRS group, PRS low = PRS medium < PGS high and PRS low < PRS high at *p* < .005. Overall significant group differences (comparing high, medium, low PRS groups) were reported for all outcomes except alcohol abuse/dependence rates, hypertension, or on high blood cholesterol (at *p* < .005). Low and high ADHD PRS groups differed significantly (after Bonferroni correction) on all outcomes with exception of alcohol abuse/dependence rates, hypertension, or on high blood cholesterol. In some cases, the low PRS group differed significantly from the medium PRS group, suggesting a protective role for low PRS scores. Low PRS group had higher cognition and education attainment and lower BMI than medium PRS group. These same variables significantly distinguished the medium and high PRS groups, as did drug abuse/dependence, ever being arrested, and perceived stress. Bonferroni corrected significance threshold of *p* < .005 applied throughout. Secondary analyses demonstrated consistent results in European- ancestry subsample of total sample.
ADHDt	5. Burton *et al.* 2019^33^	Spit for Science sample, USA N = 5,154 (comprising n = 4,426 participants with parent report; n = 728 with self report), age range: 6–17 y, (mean: 11.0, SD: 2.8). Of total sample, n = 379 had community ADHD diagnosis 49% female, 51% male Population sample European Ancestry	PRS calculation based on 10 pT (0.00001 – 0.5)	Strengths and Weaknesses of ADHD Symptoms and Normal behavior rating scale (SWAN) score^80^ total, inattentive, and hyperactive/impulsive subscales. Divided sample into low, medium, and high SWAN- scoring groups (low: z score < –1.11, n = 670; medium: z-score –0.11 to 1.11, n = 3,745, and high: z- score <1.11, n = 739). Also categorized sample using cutoff identified in ROC analyses and published cut-off of z-score >1.65. Covariates: age, sex, array, PCs	ADHD PRS was significantly associated with SWAN total score (b = 0.005, *p* = 1.7 x 10−11, R^2^ = .009), separately for parent-report (b= 0.0045, *p* = 9.0 x 10−9, R^2^ = .009) and self-report (b = 0.042, *p* = 6 x 10−4, R^2^ = 0.016) and separately for inattentive (b = 0.004, *p* = 1.6 x 10−10, R^2^ = 0.008) and hyperactive/impulsive subscales (b = 0.004, *p* = 1.3 x 10–9, R^2^ =0 .007). The association with the total score was still significant after excluding individuals with an ADHD community diagnosis. Comparisons of ADHD PRS in the categorized SWAN-scoring groups showed low < high, medium < high, but low = medium. ADHD PRS was also significantly higher when comparing groups scoring above vs. below the optimal cut-off identified in ROC analyses for parent-reported SWAN and using the Swanson cut- point of z-score >1.65. The self- rated subsample did not show a significant difference between groups. Significance threshold corrected for multiple testing throughout.
EA	6. Gialluisi *et al.* 2019^34^	Multiple samples of children with developmental dyslexia and either unrelated controls or siblings. From 8 European countries and USA (N = 2,562 –3,468) 41% female, 59% male Clinical sample European ancestry	PRS calculation based on 12 pT (0.01–1)	Diagnoses based on school history of reading problems, word reading tests, or dyslexia diagnosis. Eight outcomes relating to word reading, spelling, rapid naming, and phonology that are considered core deficits in dyslexia: word reading (WRead), nonword reading (NWRead), and word spelling (WSpell), phoneme awareness (PA), digit span (DigSpan, a measure of verbal short-term memory), and rapid automatized naming of letters (RANIet), digits (RANdig), and pictures (RANpic) Covariates: PCs	ADHD PRS was negatively associated with WRead, Wspell, and NWRead (R^2^ = 0.004–0.007%, *p* ~ [10−5–10^−7^]). ADHD PRS was not significantly associated with the other 5 outcomes. A significance threshold of 6.94 x 10^−5^ was applied to correct for multiple testing due to multiple other PRS being tested in parallel.
SES	7. Rietveld and Patel 2019^35^	Longitudinal data from the Health and Retirement Study (HRS), USA N = 9033 including participants and spouses, age range: 50–65 y 54% female, 46% male Population sample European Ancestry	No pT applied	Six later-life US labor market outcomes: currently working for pay, individual earnings (gross individual income), total household wealth (net value of total wealth, excluding second home, if applicable), receiving governmental assistance in the form of social security disability insurance, receiving unemployment or workers' compensation, receiving other governmental transfers Educational attainment included as mediator and measured by years of education Covariates: sex, age, marital status, number of living children, self-reported health, whether health limits work, tenure in current occupation, log of spousal earnings, PCs	ADHD PRS was significantly associated with all 6 labor market outcomes. A 1-SD increase in ADHD PRS associated with decrease in employment likelihood (10.15% lower odds), lower gross individual income (15.80%), and lower household wealth (12.98%). Higher ADHD PRS associated with increased likelihood of receiving social security disability benefits (20.56% higher odds), receiving unemployment or worker compensation (6.72% higher odds), and receiving governmental transfers (27.38% higher odds). For all 6 outcomes, some of the association was reduced when educational attainment was added as a mediator. Most results were highly consistent when split by sex and when split by assessments conducted at ages 50 – 55 and 50 – 59 y. A significance threshold of *p* < .05 was applied.
ADDICTION	8. Piasecki *et al*. 2019^36^	National Longitudinal Study of Adolescent to Adult Health, USA N = 5,215 unrelated participants, age range 24–34 y. Sex ratio not provided. Population sample European, African, Hispanic and East Asian Ancestry. Genetic ancestry was strongly correlated (r = 0.89) with self-identified race/ethnicity. The self-identified race/ethnicity of 9,129 individuals was n = 5754 (63%) non- Hispanic White, n = 1,940 (21%) non-Hispanic Black, n = 961 (11%) Hispanic, 449 (5%) Asian and n = 23 (<1%) Native American	PRS calculation based on pT = 1	Gambling behavior and disordered gambling The 2 phenotypes were categorical: answering yes or no to "Have you ever bought lottery tickets, played video games or slot machines for money, bet on horses or sporting events, or taken part in any other kinds of gambling for money?"; and (if "yes" to the previous question), answer of yes or no to: "Has your gambling ever caused serious financial problems or problems in your relationships with any of your family members or friends?" Covariates: age, sex, PCs	ADHD PRS was not associated with either gambling behavior or disordered gambling. A significance threshold of *p* < .05 was applied.
ASDt, NEUROPSYCH	9. Torske *et al.* 2019^37^	BUPGEN network, Norway N = 176 participants referred to a specialized hospital unit for evaluation of autism spectrum disorders (ASD), age range 5–22y with Full Scale Intelligence Quotient (IQ) >70. Most (68%) had ASD. 24% female, 76% male Clinical sample European ancestry	PRS calculation based on pT = 0.1	Diagnosis based on Autism Diagnostic Observation Schedule (ADOS), and/or the Autism Diagnostic Interview –Revised (ADI-R) Three executive function outcomes from the Behavior Rating Inventory of Executive Function (BRIEF),^81^ an 86-item questionnaire. The Behavior Regulation Index (which incorporates 3 subscales: inhibit, shift, and emotional control) and the Metacognition Index (which incorporates 5 subscales: initiate, working memory, plan/organize, organization of materials, and monitor). The Global Executive Composite Index comprised all 8 of the above subscales. Social function was assessed using the Social Responsiveness Scale, a 65-item questionnaire.^82^ Covariates: age, sex, PCs	ADHD PRS not associated with the any of the executive function outcomes or the autistic trait scale in a regression or when comparing high vs. low ADHD PRS scoring groups (those in the top and bottom 15% of the PRS distribution, respectively). Significance threshold of *p* < .05 was applied.
ADHD, ADHDt, EXTERNALISING	10. Nigg *et al*. 2020^38^	Community recruited children, USA ADHD sample: n = 337 participants, 28% female, 72% male Controls: n = 177 participants, 46% female, 54% male Age range 7 –11 y Community sample enriched for children with ADHD Northern European Ancestry	PRS calculation based on 7 pT (5 × 10^−8^– 1)	A diagnostic evaluation using standardized, well-normed rating scales from parent and teacher, parent semi-structured clinical interview, child intellectual testing, and clinical observation. Best-estimate research diagnoses and final eligibility were established by 2 experienced clinicians (a child psychiatrist and a child psychologist), who independently assigned final diagnoses. Dimensional score on an ADHD latent variable captured from hyperactivity and inattention subscales of 4 published ADHD scales Irritability captured with latent variable based on 2 subscale scores: anger and modified soothability from the Temperament in Middle Childhood Questionnaire (TMCQ)^83^ and an oppositional defiant disorder irritable total ^84^ score Latent variables were also created for surgency-approach and sadness-anxiety A person-centered approach compared different group definitions of ADHD with and without irritability and emotion dysregulation Covariates: sex, age, lifetime mood disorder and PCs	Using a structural equation model, it was shown that the ADHD PRS was associated with ADHD severity (b = 0.171, 95% Cl = 0.085–0.258; **Δ** R^2^ = 0.029, *p* < .0001), irritability (b = 0.183, 95% Cl = 0.087–0.280; **Δ** R^2^ = 0.034, *p* < .0002) and also with surgency/sensation seeking (B = 0.146, 95% Cl = 0.052–0.240, **Δ** R^2^ = 0.022, *p* = .002). These associations had adjusted for the major depression PRS85 and for the sadness/anxiety scores and their association with ADHD. The ADHD PRS was not associated with the sadness/anxiety latent variable. In the person-centered analyses (ie, looking at ADHD subgroups), the ADHD PRS was elevated in the ADHD vs. not ADHD group (OR = 1.43, 95% Cl = 1.17–1.75, **Δ**R^2^ =.033 *p* = .0004). The emotion dysregulation ADHD group had elevated ADHD PRS vs. other ADHD children (OR = 1.44, 95% Cl = 1.03–2.20, Nagelkerke **Δ** R^2^ = 0.013, *p* = .033) but the ADHD PRS did not differentiate irritable or other ADHD profiles. All effects were independent of variation in ADHD severity across traits or groups. Sensitivity analysis suggested changes in latent variable indicators or covariate handling did not influence results. Significance threshold of *p* < .01 was applied.
EA, MH	11. Dickinson *et al.* 2019^39^	National Institute of Mental Health Clinical Center, USA N = 540 participants with *DSM-IV* schizophrenia disorders, mean age 34.1 y (SD 10.1). 24.6% female, 75.4% male N = 247 siblings with no history of psychotic disorder (limited to 1 per family), 52.6% female, 47.4% male. N = 844 community control participants, 53.8% female, 46.2% male Clinical sample European ancestry	PRS calculation based on 10 pT reduced to a single score through principal components. Analyses repeated with the 10 pT (0.0001–0.5)	Participants with schizophrenia and their siblings were assigned to 1 of 3 clusters based on trajectories of cognitive development: cognitively stable (CS), adolescent decline (AD), preadolescent impairment (PI). Wide-Range Achievement Test [WRAT] reading subtest^86^ and Wechsler Adult Intelligence Scale [WAIS]^87^ used for cognitive assessments. Covariates: sex, age, PCs	The ADHD PRS did not differ significantly between schizophrenia patients, siblings and controls. Within the participants with schizophrenia, the ADHD PRS showed significant association with cognitive trajectory group (F = 5.1 df = 2,525, *p* = .007, R^2^ = 0.019%). Pairwise comparisons showed PI > AD = CS (at *p* < .05). Within the siblings, the ADHD PRS did not show a significant association with cognitive trajectory group (F = 0.3 df = 2,232), and no pairwise comparisons were significant at *p* < .05.
ADDICTION	12. Cabana-Domínguez *et al.* 2019^40^	SAGE (USA) and 3 other dbGAP sample datasets; N = 2,083 cases, age range unknown, 41.6% male N = 4287 controls 44% female, 56.0% male Clinical sample European ancestry	PRS calculation based on 9 pT (1 × 10^−4^ – 1) reduced to single score with PCA	Cocaine dependence, as measured by the *DSM-IV*^75^	ADHD PRS was significantly associated with cocaine dependence (pseudo- R^2^ = 1.39%, *p* = 4.5e__17_). SNP threshold of *p* < 5.7e−04 applied to account for multiple testing
MH	13. Ohi *et al.* 2020^41^	The Schizophrenia Non-Affected Relative Project, Japan N = 332 participants, n = 130 patients with schizophrenia, 38.2% female, 61.8% male, mean age = 42.9, SD = 13.1 y n = 56 unaffected first-degree relatives (41 parents/12 siblings/4 offspring), 68.4% female, 31.6% male, mean age: 59.7, SD: 13.6 y n = 146 controls, 33.3% female, 66.6% male, mean age: 37.2, SD: 14.1 y Clinical sample Japanese descent	PRS calculation based on 6 pT (0.01–1)	Schizophrenia (based on the criteria of the *DSM-5*^74^ or being a first-degree relative of someone with schizophrenia Covariates: PCs	ADHD PRS were not significantly different between all the groups (patients with schizophrenia, their first-degree relatives, and controls) or between any pairwise comparisons at *p* < .01. Significance threshold of *p* < .01 applied to correct for multiple testing.
BRAIN	14. Mooney *et al.* 2020^42^	N = 312 Participants, age range: 7–15 y (mean age: 10.2 y), USA ADHD sample: n = 199 (30% female, 70% male); control sample: n = 113 (47% female, 53% male) Community sample enriched for ADHD Northern European ancestry	PRS calculation based on pT = 0.5	Diagnosis by Conners' Rating Scales –3rd Edition short form, Strengths and Difficulties Questionnaire long form including the impairment module (SDQ), the ADHD Rating Scale ADHD-RS MRI: total brain volume (TBV) and subcortical structures Covariates: motion during MRI scan, PCs, age, sex, average FD (ie, motion during the scan [average framewise displacement]), sex interaction effect, diagnosis; TBV also a covariate in analyses on subcortical structures	ADHD PRS was negatively associated with TBV (β = −0.147 [−0.27 to –0.03]), and this remained significant after controlling for ADHD diagnosis. TBV accounted for 16% of the association between ADHD PRS and ADHD diagnosis after accounting for sex and age. ADHD PRS was not significantly associated with subcortical brain structures Among females only, the ADHD PRS was significantly associated with increased putamen volume (β = 0.224 [0.09–0.36]) FDR correction (α = 0.05) for the 9 volumes tested
ADHD, ADHDt, ADDICTION, ASDt, EA, EXTERNALISING, NEUROPSYCH	15. Vuijk *et al.* 2019^43^	Longitudinal Study of Genetic Influences on Cognition (LOGIC) N = 433 participants, age range 7 – 18 y, mean age: 11.5, SD: 3.1 y. Clinical sample with wide range of diagnoses including ADHD. ADHD participants compared to individuals with other *DSM-IV* Axis 1 diagnoses 37.2% female, 62.8% male Clinical sample Second sample for replication: N = 5,140, 19-to 60-year-old adult patients from a local health system biobank European ancestry	PRS calculation based on 10 pT	*DSM-IV*^75^ Axis 1 diagnoses; a range of parent-rated dimensional published scales of psychopathology Somatic complaints measured with the CBCL^76^ Social cognition measured with the SRS^82^ IQ and working memory from the Wechsler Intelligence Scale for Children–Fourth Edition for 7- to 16-year-olds and the Wechsler Adult Intelligence Scale–4th Edition 17- to 18- year-olds ^87^,^88^ Academic achievement with the Word Reading and Numerical Operations of the Wechsler Individual Achievement Test -Third Edition^89^ (WIAT-III). The adult replication cohort outcomes were /CD-10 ADHD, whether education was completed by age 23 y or not, and presence of substance use disorder history. Covariates: age, sex, genotyping wave (in biobank analyses), PCs	In this clinical sample including a wide mix of psychiatric diagnoses, ADHD PRS was associated with broad ADHD diagnosis (OR = 1.44, 95% Cl = 1.14–1.81; Pseudo R^2^ = 2.01; permuted *p* = .0011) as well as ADHD traits (b = 1.46; R^2^ = 2.93%; F = 11.83, permuted *p* = .0007) and with Hyperactivity/Impulsivity subscale (b = 0.97; R^2^ = 2.00%; F = 8.81, permuted *p* = .0063) but not with Inattention. For non-ADHD outcomes, the ADHD PRS predicted word reading (b = −2.11; R^2^ = 2.05%; F = 8.68, permuted *p* = .0043) and numerical operations (b = −2.20; R^2^ = 2.27%; F = 9.25, permuted *p* = .0030). ADHD PRS was also associated with aggressive behavior (b = 1.58; R^2^ = 2.59; F = 10.52, permuted *p* = .0019) and working memory index (b = −2.17; R^2^ = 2.47; F = 10.10, permuted *p* = .0016). Controlling for ADHD and stimulant use did not change the above non-ADHD outcome findings. ADHD PRS did not significantly predict somatic complaints measured with the CBCL^76^ or social cognition measured with the SRS,^82^ considered to demonstrate discriminant validity of the ADHD PRS. Results are reported for the most significant pT. The adult psychiatric sample showed similar results. ADHD PRS was associated with ADHD diagnosis (OR = 1.21, 95% Cl = 1.07–1.37, Pseudo R^2^ = 0.42%, *p* = .0028) reduced likelihood of college completion (OR = 1.23, 95% Cl = 1.12–1.35, Pseudo R^2^ = 0.72%, *p* < .0001) and substance use disorder (OR = 1.18, 95% Cl = 1.10–1.26, Pseudo R^2^ = 0.40%, *p* < .0001). Division of youth sample into high (>30%), medium (middle 40%), and low (<30%) PRS scoring groups showed that the high group had a more severe multivariate pattern of psychopathology compared to the low group (b = 0.21, *p* =.01). No significant differences between the medium and low groups were found Bonferroni correction for multiple outcomes
ADHDt, EXTERNALISING	16. Li 2019b^44^	National Longitudinal Study of Adolescent to Adult Health (Add Health), USA N = 7,674 participants, age 7– 12 (wave 1) age range 18–32 y (later waves). 54% female, 46% male Population sample 63.2% Caucasian, 21.2% African- American, 5.1% Asian, and 10.6% Hispanic.	PRS calculation based on pT = 1	Latent classes were derived for externalizing behaviors (which included aggressive behaviors, nonaggressive rule-breaking, and substance use behaviors) assessed at waves 3 and 4 by in-person interviews 4 mediators selected from wave 1 assessment: Supportive parenting, school connectedness and sensation seeking assessed with questionnaires; Peer closeness assessed in relation to 10 named friends ADHD assessed retrospectively with *DSM-IV* items at Wave 3 Covariates: PCs, sex, age, highest level of education, income	ADHD PRS correlated 0.084 with ADHD symptoms (*p* < .01) ADHD PRS predicted 17.0% increased odds in the High Decreasing (OR = 1.17 95% Cl = 1.002, 1.366, *p* = .05) and 8.0% increased odds in the Moderate (OR = 1.08, 95% Cl = 1.004, 1.163, *p* =.03) externalizing trajectories, but was not associated with the Low Increasing (95% Cl = 0.868, 1.265) trajectory, relative to the Normal trajectory group There was no longer evidence of direct associations between ADHD PRS on externalizing trajectory groups relative to the Normal trajectory group once mediators were added to the models. School connectedness either partially or fully mediated the effects Significance threshold was *p* <.05
EXTERNALISING	17. Riglin *et al.* 2019^45^	Avon Longitudinal Study of Parents and Children (ALSPAC), UK N = 7924 participants, age range 7–15 y Population sample European ancestry	PRS calculation based on pT = .05 in primary analyses; analyses repeated on multiple thresholds	Growth mixture modeling gave 5 distinct irritability trajectory classes: low, decreasing, increasing, late-childhood limited, and high-persistent Parent-reported data on irritability from the oppositional defiant disorder section of the Development and Well-Being Assessment (DAWBA),^1^ a structured research diagnostic interview, at ages 7, 10, 13 and 15 y DAWBA was also used to diagnose ADHD, oppositional defiant disorder, conduct disorder, generalized anxiety disorder, and depression.	ADHD PRS was associated with an increased likelihood of being in both the high-persistent (OR = 1.31, 95% Cl = 1.09–1.58, *p* = .005) and the increasing (OR = 1.28, 95% Cl = 1.11–1.48, *p* = .001) trajectory classes relative to the low irritability trajectory class. The odds were similar for being in either trajectory (high-persistent compared with increasing trajectory class: OR = 1.02, 95% Cl = 0.81–1.29, *p* = .854). The ADHD PRS did not predict being in the decreasing or late childhood limited trajectory groups. Results were consistent when sex was controlled for and when individuals with diagnoses were excluded. PCs were not controlled for. Significance threshold was *p* < .05.
ADHD, MH, OTHER	18. Grigoroiu-Serbanescu *et al.* 2019^46^	Romania and UK case-control samples Romanian sample: N = 470 bipolar disorder (BP) cases (all BP type 1) (60% female; 40% male 2%); 329 controls (57% female; 43% male); 43% of BP cases have childhood ADHD. UK sample: N = 472 BP cases with childhood ADHD data (67% BP type 1, 33% BP type 2) (65% female; 35% male) and 1,287 controls (34% male; 66% female). 34% of the BP cases has childhood ADHD. Romanian and UK sample results were meta-analyzed. Clinical sample European ancestry	PRS calculation based on 10 pT (0.01–0.5)	Bipolar disorder in the UK sample was assessed using the ICD-10, and in the Romanina sample with *DSM-IV*^75^ criteria, based on Diagnostic Interview for Genetic Studies (DIGS) and medical records. Childhood ADHD within BP cases was assessed retrospectively using the Wender Utah Rating Scale (WURS)^90^ and for some Romanian cases also using items from the Kiddie-SADS^91^ clinical interview. Assessment of childhood ADHD was made by clinicians. Early- and late-onset BP defined as age of onset at >22 y or >22 y of age, respectively. No covariates	ADHD PRS differentiated BP cases with childhood ADHD from controls in the meta-analysis of both samples (OR = 0.2 (0.08 −0.32), z =3.23, FDR-corrected *p* = 0.024). The ADHD PRS differentiated BP cases with childhood ADHD from BP cases without childhood ADHD in the meta-analysis, but this did not survive FDR correction (OR = 0.18 (0.04–0.31), z = 2.55, *p* = .011 FDR-*p* = .055). ADHD PRS associated with the continuous measure of ADHD symptoms (based on WURS and Kiddie-SADS) within the BP cases in the meta-analysis (b = 1.7 (0.7 –2.69), z = 3.34, *p* = 0.0008, FDR- corrected *p* = 0.024). This result remained when sex or BP age of onset were included as covariates. This association was found to be driven by BP cases with early onset (<22 y). ADHD PRS did not differentiate all BP cases from controls at either nominal or FDR-corrected significance (OR = 0.085 (0 − 0.17), z = 1.95, *p* = .051, FDR-corrected *p* = .105). However, it did differentiate early-onset BP cases from controls (OR = 2.51 (1.04 –3.97), z =3.36, *p* = .0008, FDR- corrected *p* = .024) but not late- onset cases. ADHD PRS predicted earlier age of onset within BP group (b = −0.92, (−1.61 to 0.23), z = −2.62, *p* = .009, FDR-corrected *p* = .049). Results given here for most significant PRS pT. FDR correction was used to adjust significance for multiple testing.
ADDICTION, EXT, OTHER, SES	19. Wimberley *et al.* 2019^47^	IPSYCH Sample, Denmark, born 1981–2003. N = 13116 participants with ADHD, 26% female, 74% male Of these, n = 2368 (18.1%) developed SUD (27% female, 73% male). Median age at first SUD diagnosis was 19.4 y (IQR 17.2–22.3 y). Clinical sample from population cohort Due to overlap with Demontis et al^17^ discovery sample, participants split into 5 groups, with each group consecutively used as target sample, and the remaining 4 groups plus other Psychiatric Genomic consortium samples as the discovery sample. European ancestry	PRS calculation based on pT 0.2	At least 1 substance use disorder (/CD-8 and ICD-10– Diagnostic Criteria for Research [DCR]^23^) in Danish registers after 13^th^ birthday. Categorized by type into alcohol, cannabis, and other illicit drugs, and second categorized into severity into use, abuse, and addiction. Nicotine use not included. Other known SUD risk factors (presence of comorbid oppositional defiant disorder/conduct disorder (ODD/CD), parental SUD, parental mental disorder, paternal income, maternal education, obtained from IPSYCH and Danish registers Covariates: observation time (to account time at risk for SUD given varying ages of participants), sex, age. and calendar year at first ADHD diagnosis and PCs	ADHD PRS were associated with any SUD (OR = 1.30, 95% Cl = 1.11 −1.51; Nagelkerke R2= 0.14). For types of SUD, associations were observed for alcohol (OR = 1.26, 95% Cl = 1.04–1.53), cannabis (OR = 1.34, 95% Cl = 1.10–1.64) but not illicit drugs (OR = 1.21, 95% Cl = 0.99–1.50). For severity of SUD, associations were observed for use (OR = 1.36, 95% Cl = 1.02–1.80) and addiction (OR = 1.30, 95% Cl = 1.07–1.57) but not abuse (OR = 1.21, 95% Cl = 0.88–1.65). Stratified by sex, the point estimate for the ADHD PRS-SUD association was higher in females, but CIs overlapped with CIs for males. The other known SUD risk factors were all themselves associated with ADHD PRS (at *p* < .001). Nevertheless, the above SUD associations still remained with the ADHD PRS when controlling for these known SUD risk factors. Sensitivity analyses repeated with different pT, different assumed prevalences of ADHD and SUD, and variation in population structure showed similar results. Significance threshold was Bonferroni corrected to *p* < .007.
MH	20. Riglin *et al.* 2020^48^	Avon Longitudinal Study of Parents and Children (ALSPAC), UK N = 5,518 at age 7 y and N = 7,017 at age 13 y Population sample European ancestry	PRS calculation based on pT <.05 in primary analyses; repeated on multiple thresholds	A "general psychopathology" ("p") factor for ages 7 and 13 y Emotional, behavioral and neurodevelopmental problems were determined with the DAWBA.^72^ Additionally, the Social and Communication Disorders Checklist^92^ (SCDC) was used for social-communication problems related to ASD. No covariates	ADHD PRS was associated with the general psychopathology "p" factor at age 7 (B = 0.087, SE = 0.019, *p* < .001), and age 13 (B = 0.095, SE= 0.020, *p* < .001) while including the above other 3 PRS in the models. Without other PRS in the model, the ADHD PRS predicted the p factor at age 7 (B = 0.093, SE = 0.019, *p* < .001, R^2^ = .009%) and age 13 (B = 0.095, SE = 0.019, *p* < .001, R^2^ = 0.009%) Results were consistent when the other PRS were excluded from the model, and analyses repeated using inverse probability weighting to address potential bias due missing genetic data revealed similar results, as did analyses at other pT.
BRAIN, EXTERNALISING, PHYSICAL	21. Barker *et al.* 2019^49^	IMAGEN Study, France, UK, Ireland, Germany N = 604–874 participants Population sample European ancestry	PRS calculation based on pT 0.05	BMI derived from height and weight measurements at age 19 y Voxel-based morphometry measures of whole-brain gray matter at age 19 y Neural responses to reward anticipation and reward outcome from activation maps from a Monetary Incentive Delay fMRI task at age 19 y A neural endophenotype created which was made up of gray matter regions and regions of activation derived from the fMRI task. Impulsivity symptoms at age 19 assessed using self-reported Barratt Impulsivity Scale (BIS)^93^ Covariates: sex, imaging site, age, PCs, and total intracranial volume	ADHD PRS correlated with impulsivity symptoms (r = 0.10, *p* = .014 FWE corrected). ADHD PRS was correlated with the neural endophenotype (r = 0.087, *p* = 0.036 FWE corrected). In mediation analyses, the ADHD PRS associated via the neuroimaging substrate with impulsivity symptoms (b = 0.006, 90% Cl = 0.001, 0.019) and BMI (b = 0.009, 90% Cl = 0.001, 0.025). Significance levels ascertained from permutation testing and 1-sided tests, and corrected for multiple testing.
ADHDt, EA	22. De Zeeuw *et al.* 2019^50^	The Netherlands Twin Register (NTR) Trios (ie, 1 offspring and both parents). N = 1120–2518 Population sample European ancestry	PRS based on transmitted and non-transmitted alleles for 8 pT (0.0001 –0.5)	ADHD symptoms (CBCL and Teacher Report Form (TRF) Attention Problems scale^76^) were assessed at age 10 or 12 y. Academic achievement was assessed with the Cito score, a Dutch nationwide standardized educational achievement test.^94^ Educational attainment in adults was assessed as self-reported highest degree. Covariates: sex, year of birth (only for EA), the interaction between sex and year of birth (only for EA), PCs, genotyping platform	EA PRS and ADHD PRSs correlated for both the transmitted and non transmitted PRS (r = − 0.27 and r = − 0.23, respectively). ADHD transmitted and non transmitted PRS were not significantly associated with academic achievement (R^2^ ~ 0.6%). ADHD transmitted PRS was associated with ADHD symptoms (R^2^ = 1 %–2%). The transmitted ADHD PGS was associated with ADHD symptoms at home (ß = 0.17, Cl 0.12–0.21, R^2^ = 2.7%, *p* = 2 x 10−13) and at school (ß = 0.13, Cl = 0.08–0.17, R^2^ = 1.6%, *p* = 3 x 10−7) but not with academic achievement (ß = - 0.08, Cl = −0.14–0.01, R^2^ = 0.6%, *p* = .022). In a model that included both the EA PRS and ADHD PRS, the above effects remained between ADHD PRS and ADHD symptoms at home and school, but the association between ADHD PRS and academic achievement was no longer significant. The non-transmitted ADHD PGS was not associated with any of the above 3 outcomes. Significance threshold of *p* < .01 used.
MH	23. Yao *et al.* 2019^51^	Child and Adolescent Twin Study in Sweden (CATSS) N = 13,472 participants, assessed at age 15 y Population sample European ancestry	PRS calculation based on pT <1 for primary analyses, and on 7 pT (0.00001–1) for sensitivity analyses	Self-reported Eating Disorder (ED) symptoms were measured by 3 subscales (Drive for Thinness, Dissatisfaction) from the Eating Disorder Inventory −2 (EDI-2)^95^ at 15 y Covariates: sex, birth year, and PCs	ADHD PRS was associated with the EDI-2 full scale (b = 0.027, 95% Cl = 0.005, 0.049, R^2^ = 0.0012%, *p* = .015) and subscales Drive for Thinness (b = 0.032, 95% Cl = 0.005, 0.059, R^2^ = 0.0010%, *p* = .022) and Body Dissatisfaction (b = 0.042, 95% Cl = 0.011, 0.072, R^2^ = 0.0013%, *p* = .007) but not the Bulimia subscale (b = 0.004, 95% Cl = −0.013,0.021, R^2^ = 0.0000%, *p* = .654). Results were consistent at other pT; sex differences were not significant. Significance threshold was *p* < .05.
ADDICTION, OTHER	24. Rabinowitz *et al.* 2018^52^	Sample from urban school district in the mid-Atlantic region of USA N = 1,050 participants 56% female, 44% male Population sample African American	PRS calculation based on pT < 0.05	To assess past-year marijuana abuse and dependence at age 20 y, Composite International Diagnostic Interview –University of Michigan Version (CIDI-UM)^96^ was used in 2 cohorts. In the third cohort, National Survey on Drug Use and Health (NSDUH)^97^ was used. The Structured Interview of Parent Management Skills and Practices–Youth Version (SIPMSP)^98^ was used to assess parental monitoring (proximal contextual factor). The community disadvantage score was calculated using census-tract level items from the 1990 and 2000 Decennial census^99^ (distal contextual factor). Covariates: PCs	The ADHD PRS correlated negatively with parental monitoring (r = −0.07, *p* < .05) but was not significantly correlated with community disadvantage (r = −0.04, *p* > .05). ADHD PRS was not associated with marijuana use disorders and the ADHD PRS x community disadvantage and ADHD PRS x parental monitoring interactions were also not significant, nor were 3-way interactions involving sex, ADHD PRS, and either community disadvantage or parental monitoring. Significance threshold was *p* < .05.
ADHDt	25. Taylor *et al.* 2019^53^	Child and Adolescent Twin Study in Sweden (CATSS) N = 13391 participants 50% females, 50% male Population sample European ancestry	PRS calculation based on pT 0.5. Analyses repeated on 5 other pT	ADHD traits were measured with The Autism–Tics, ADHD and Other Comorbidities inventory (A-TAC)^100^ assessed by parents at ages 9 and 12 y Covariates: sex, age, PCs	ADHD PRS was associated with ADHD traits at ages 9 and 12 y (β [SE] = 0.27 [0.03], R^2^ = 8.4 × 10^−3^, *p* = 5.9 x 10^−19^) and ADHD trait subscales hyperactivity/impulsivity (ß [SE] = 0.14 [0.02], R^2^ = 7.7 x 1CT3, *p* =1.9 x 10^−19^) and inattention (ß [SE] = 0.13 [0.02], R^2^ = 6.0 x 10^−3^, *p* = 2.9 x 10^−15^). After excluding children with *ICD-10* diagnosed ADHD, ADHD PRS was still associated with ADHD traits (β [SE] = 0.21 [0.03], R^2^ = 6.2 x 10^−3^, *p* = 2.2 x 10^−13^) and the ADHD subscales. FDR-corrections was applied to adjust for multiple testing.
ADHDt, BRAIN	26. Alemany *et al.* 2019^54^	Generation R Study, The Netherlands N = 1,053 –1,139 participants, the mean age: 10.16 y, SD = 0.60, age range = 8.72–11.9 y 49% female, 51% male Population sample European ancestry	PRS calculation based on 6 pT "priors" (0.01 - infinitesimal)	Structural MRIs; Image processing using FreeSurferto extract cortical and subcortical brain volumes. Ten volumetric brain measures used as outcomes: total brain volume (TBV), cortical gray matter (GM), total white matter, subcortical GM, ventricular volume, cerebellum, amygdala – hippocampus complex, caudate, putamen, and thalamus (final 3 are subcortical brain volumes) Assessed on CBCL^76^ attention problems subscale at ages 8–11 y Covariates: sex, age, total intracranial volume (for all except TBV analysis), PCs	ADHD PRS was associated with attention problems subscale (b = 0.12, SE = 0.00, *p* = 5.36 x 10^−5^). ADHD PRS was associated with smaller caudate volume (result for strongest prior: (b = −0.08, SE = 0.03, P_uncon-ected_ =7.49 X 10^4^) across all priors except prior 1 at *p* < .05 and 1 prior was significant after FDR correction. In subsequent mediation analyses, no evidence of caudate volume acting as a mediator between ADHD PRS and attention problems in full sample. Stratified by sex, mediation was significant for boys, indicating that 11% of the association between ADHD PRS (prior 0.01) and attention problems was mediated by differences in caudate volume. ADHD PRS was associated with smaller TBV (result for strongest prior: β = −0.07, SE = 0.03, *p*_uncorrected_ = −006) across all priors except prior 0.01 at *p* < .05, but none significant after FDR correction. FDR correction at *p* < .05 used as significance threshold.
ADDICTION	27. Gurriaran *et al.* 2018^55^	Sample from the Addictive Disorders Assistance Units from Galicia health care areas, Spain N= 534 substance abuse/ dependence patients (mean age 44.89 y, SD 9.73) 13% female, 87% male N = 587 Control subjects recruited from blood donors at Santiago de Compostela, Galicia. Mean age 40.26 y (SD = 10.70; range = 18–65). Not checked for substance use 50% female, 50% male Clinical sample European ancestry	PRS calculation based on 6 pT (0.001–1)	DSM-/V^75^ criteria for substance use disorder Covariates: sex, age, PCs	ADHD PRS was not associated with substance use disorders after multiple testing correction (P_seudo_ R^2^ = ~0.4, *p* < .05, *p* > .002). Results were similar when MHC was included. Permutation based *p* value of *p* < .0022 used.
BRAIN	28. Szekely *et al.* 2018^56^	The LONG Cohort, USA n = 119 cases, n = 339 controls Mean age at first scan = 11.47 y, SD = 3.54; mean age at second scan = 16.13 y, SD = 4.72. 41% female, 59% male Population sample enriched for ADHD cases n = 404 European Americans, n = 31 African Americans, n = 8 Asian Americans, and n = 15 participants of mixed race	PRS calculation based on 7 pT (0.0005 – 0.5)	association between polygenic risk for ADHD and brain growth was determined for the LONG cohort association between polygenic risk for ADHD and brain growth was determined for the LONG cohort ADHD ascertained using clinician-administered Parent Diagnostic Interview for Children and Adolescents^107^ Longitudinal growth in volume across 2 time points modeled linearly for 4 brain divisions: cerebral cortex, basal ganglia, cerebellum, cerebral white matter, and 1 region of interest: right lateral prefrontal cortex Covariates: adjusted for age at baseline scan, interscan interval, sex, and PCs	ADHD PRS was not associated with any brain growth phenotypes (all *p* > 0.1). Significance threshold was not reported.
ADHD, ADHDt, NEUROPSYCH	29. Nigg *et al.* 2018^57^'^101^-^129^	Community recruited sample, USA Full sample N = 656 European-only sample n = 514 (337 ADHD, 71% male; 177 non- ADHD, 52% male) age range: 7–11 y 22% non-European, 78% European ancestry Community sample enriched for children with ADHD	PRS calculation based on pT 0.5 Results checked for another 6 pT	(a) Stop-Go task^107^; (b) Identical Pairs Continuous Performance Task^108^; (c) Spatial span forward and backward^109^; (d) Digit span forward and backward from the Wechsler Intelligence Scale for Children, Fourth Edition^110^; (e) N-back, including 0-back, 1- back, and 2-back conditions; (f) Delis, Kaplan, and Kramer (DKEF)^111^ version of the Stroop task (word, color, and color-word); (g) DKEF Trailmaking test (number, letter, and shifting); and (h) a motor time reproduction task at fast (500- millisecond) interval from which we derived clock precision (Parclock variation)^112^. ADHD diagnoses made using *DSM-IV* criteria and a best- estimate procedure Separate parent and teacherrated ADHD symptom latent variables derived from data on 3 or 4 published ADHD measures that capture inattention and hyperactivity Cognitive latent variables were captured using PCA models from data on laboratory measures of working memory, response inhibition, executive functioning, arousal/attention, temporal information processing, and processing speed^124^–^129^ Covariates: sex, age, PCs	ADHD PRS was associated with ADHD diagnosis (Nagelkerke R^2^ =0.045%; b = 0.233, SE = 0.053, *p* = .000011) and both parent and teacher-rated ADHD symptom latent variables (R^2^ =0.033%; b = 0.185, SE = 0.043 *p* = 1.69E-05 and R^2^ =0.027%; b = 0.165, SE = 0.042, *p* = 8.55E-05 respectively). Of the 5 latent cognitive variables, ADHD PRS only predicted working memory (b = 0.227, SE = 0.040, *p* = 1.39E-08) and vigilance/arousal (b = 0.130, SE = 0.049, *p* = .0079). It did not predict slow output speed, mental clock, or response inhibition. In mediation models, the ADHD PRS effect on ADHD diagnosis was statistically mediated by working memory (indirect effect, b = 0.101, SE = 0.029, *p* = .00049, 43% of genetic effect accounted for) and arousal/ alertness (indirect effect b = 0.115, SE = 0.041, *p* = .005, 49% of genetic effect accounted for). The same was found for models with ADHD PRS predicting parent and teacher-rated ADHD symptom latent variables, with 43% – 51% of the genetic effect accounted for by the latent cognitive variables. Direct PRS tests had a Hochberg correction *p* < .05. Mediation models used *p* <.05. Analyses repeated including non- European LONG sample participants, and when changing the discovery sample to be European-only, both led to similar conclusions.
ADHD	30. Hawi *et al.* 2018^58^	Participants recruited in Australia, UK and Ireland. N = 480 ADHD case subjects aged 5 – 18 y (mean age = 10.27 y, SD= 3.03). 13% female, 87% male N = 1208 controls, age 7–60 y (mean age = 20.61 y, SD = 6.76) 51% female, 49% male European ancestry	1000 pT from 0.0005 to 0.5	The association between the ADHD PRS and variance in the ADHD case-control status. ADHD cases met the criteria of ADHD of *DSM-IV*^74^. ADHD status using *DSM-IV* criteria determined with parental semi-structured interview and the Conners' Parent Rating Scale^108^ Covariates: sex, age2 , age x gender, PCs	ADHD PRS explained 3.25% variance in ADHD case-control status (Nagelkerke' s R^2^ = 0.03, *p* = 7.6E −15) Significance threshold *p* =.001 applied.
OTHER	31. Taylor *et al.* 2018^59^	Avon Longitudinal Study of Parents and Children (ALSPAC), UK N = 7,486 mothers, N = 7,508 children Population sample European ancestry	PRS calculation based on 5 pT (0.0005 – 0.5) as well as just genome-wide significant SNPs	Nine participation phenotypes derived. Participation defined as responding to a questionnaire or attending a clinic for which the whole cohort was eligible to participate Continuous phenotypes calculated by summing the number of questionnaires/ clinics completed and or clinics attended Covariates: child sex, PCs	ADHD PRS was negatively associated with all 9 mother and children participation phenotypes. For example, ADHD PRS predicted mother total participation score negatively (ES = −2.18, 95% Cl = −2.71 to 1.64), and it predicted the child total participation score negatively (ES = −2.14, 95% Cl = −2.63 to 1.64). Significance threshold not given: results reported as effect sizes.
ADHDt, EA, PHYSICAL, MH, SES	32. Selzam *et al.* 2019^60^	Twins Early Development Study, UK N = 789–2,962 dizygotic (DZ) twin pairs, assessed from 12 to 21 y Population sample European ancestry	PRS calculation based on pT 1 (using a prior)	Parents reported on twins' ADHD traits via the Strength and Difficulties Questionnaire^73^ hyperactivity subscale and the Conners' rating scales^108^ at ages 12 and/or 16 y Educational attainments based on standardized tests taken at the end of compulsory education in the UK (General Certificate of Secondary Education [GCSE]) as obtained for twins at age 16 y BMI and height were self-reported. IQ involved verbal and nonverbal ability using WISC-III assessments Psychotic experiences assessed using the Specific Psychotic Experiences Questionnaire^109^ at age 16 y. Neuroticism assessed using a Big Five questionnaire ^110^ Self-rated health assessed using the RAND Short-Form Health Survey^111^ Socioeconomic status: based on maternal age at birth of the first child, maternal and paternal highest education level, and maternal and paternal occupation. Covariates: PCs, chip, plate, and phenotypes were corrected for age and sex	The ADHD PRS effect was split into between-family and within-family effects using DZ twin data. The between-family ADHD PRS effect, which was estimated independent of the within-family effect, significantly predicted more ADHD traits (b = 0.11, Cl = 0.08–0.14; *p* = 6.8 x 10^−9^), higher BMI (b = 0.07, Cl = 0.03–0.11; *p* = .008), lower IQ (b = −0.09, Cl = −.12 to −.05; *p* = 4.5 x 10– 4), and lower GCSEs (b = −0.18, Cl = −0.21 to −0.15; *p* = 7.3 x 10^−17^). The within family ADHD PRS effect showed that, within pairs, the twin with higher ADHD PRS had more ADHD traits than their co-twins (b = 0.12, Cl = 0.08–0.17, *p* = 1.50e^−7^). Within pairs, the twin with higher ADHD PRS also lower GCSE grades than their cotwins (b = −0.06, Cl = −0.10 to −.03, *p* = .001). The ADHD GPS within-family prediction was significantly lower than between-family prediction for GCSEs (b = −0.12, Cl = −0.16 to −0.07, *p* = 4.95e x ^−5^, Diff = 65.4%). The between-family ADHD PRS effect on GCSEs was significantly reduced when socioeconomic status was controlled for (*p* = 7.69 x e^−4^) but was still significant. The ADHD PRS also significantly predicted lower SES (b = −0.17, Cl = −0.21 to −0.13, *p* = 1.32e−13) The ADHD PRS did not significantly predict (either as within- or as between-family effect): height, self- rated health, neuroticism, psychotic experiences. Results were stable when analyses were rerun on the sample split by same-sex/opposite-sex twins, based on differences in CHIP, using a prior pT of 0.1, and using PRSs with British samples removed Statistical significance was *p* < .01, based on an Benjamini Hochberg false discovery rate (FDR) adjustment
OTHER	33. Schoeler *et al.* 2019^61^	Avon Longitudinal Study of Parents and Children (ALSPAC), UK N = 5,028 participants Assessed at age 8, 10 and 13 y 51% female, 49% male Population sample European ancestry	PRS calculation based on 99 pT (0.01–1)	Exposure to bullying was assessed based on child reports at 8, 10, and 13 y of age using a modified version of the Bullying and Friendship Interview Schedule (BFIS).^112^ Mean score of exposure to bullying across ages was used. Covariates: sex, PCs	ADHD PRS was significantly associated with bullying (standardized b = 0.085; 95% Cl = 0.056 – 0.113, *p* < .001). In a multi-PRS analysis with 10 other significant PRS predictors, ADHD PRS was still significantly associated with bullying (standardized b = 0.062; 95% Cl, = 0.032–0.092, *p* < .001). Repeated multi-PRS analysis, which looked at chronicity of bullying, showed similar results. There was no evidence of an interaction effect of sex. The multi-PRS association of ADHD PRS and bullying was no longer significant when bullying perpetration was included as a covariate. Permutation and false discovery rate–corrected p values were applied to estimate significance thresholds.
OTHER	34. Mooney *et al.* 2020^62^	Community volunteers, USA N = 472 participants: n = 302 with ADHD (72.5% male), mean age = 9.9 y (SD = 1.4); n = 170 without ADHD (54.1% male), mean age = 9.8 y (SD = 1.4) Community sample enriched for ADHD European ancestry	PRS calculation based on pT 0.5	Diagnosis based on: diagnostic parent interview (Kiddie Schedule for Affective Disorders and Schizophrenia for School-Age Children– Epidemiologic Version [KSAD-S-E]), parent and teacher standardized rating forms that assessed symptoms and impairment, clinician observations A total of 568,281 probes assessed for DNA méthylation on the MethylationEPIC BeadChip. Differential global méthylation (average methylation across all probes), as well as differentially methylated positions (DMPs) derived from saliva. Cell-type adjusted β values were the outcome variables. Covariates: sex, age, PCs, medication usage, maternal smoking, number of missing SNPs in the PRS calculation for each patient, and a sex interaction term	The ADHD PRS was associated with reduced DNA methylation at 1 probe, cg15472673 at genome-wide significance (*p* = 6.71 E^−8^), and this association remained (*p* = 9.76e^−8^) when including ADHD status in the regression model, suggesting that the effect was not driven by elevated polygenic burden in ADHD cases. The probe is located between the GART and SON genes in a CpG island of a bivariate promoter. The SNPs in the ADHD PRS are not direct methylation quantitative trait loci for cg15472673, as such the association with the PRS is not thought to be a genetic effect on DNA méthylation. The ADHD PRS was associated with DNA méthylation levels at 12 other probes at *p* < 1.0e^−5^. No sex interactions were significant at the EWAS significance threshold. In terms of differentially methylated regions, 1 region on chromosome 6 within the major histocompatibility complex was identified, in which the ADHD PRS associated with 8 probes associated with the ADHD PRS. The association was sex-specific: in female participants; a higher PRS was associated with higher méthylation levels, and the opposite was found for males.
ADHDt, BRAIN, EA, NEUROPSYCH	35. Sudre *et al.* 2018^63^,^113^	N = 544 participants (mean 21 y, 212 [39%] with ADHD). Majority European ancestry. Subpopulations with white non-Hispanic ancestry and African American ancestry. Clinical sample	PRS calculation based on 7 pT (0.01 – 0.5)	Inattention and hyperactivity disorder symptoms measured using clinician administered Diagnostic Interview for Children and Adolescents for parents.^2^ Adult symptoms of ADHD were measured by clinicians using the Conners' Adult ADHD Diagnostic Interview for *DSM-IV*.^3^ Neuroanatomic imaging, and imaging of white matter tract microstructure Other disorders in adults were ascertained through the Structured Clinical Interview for *DSM-IV*-TR Axis 1 Disorders.^4^ Working memory spans assessed through number of correctly recalled digits/tapping patterns Processing speed assessed using visual matching task (from the Woodcock Johnson III Test of Cognitive Abilities^130^) IQ was assessed using an age appropriate version of the Wechsler scales Attentional processes measured using the Conners' Continuous Performance Test,^131^ from which focused attention, perseverative/impulsive responding and sustained attention were derived. Covariates: Age, sex. Also for imaging data: motion and quality control scores	ADHD PRS predicted symptoms of hyperactivity–impulsivity (b = 0.11, SE = 0.046, *p* = .02, at FDR q < 0.05), but not inattention (at FDR q < 0.05). Of the neuroanatomic mediators (white matter microstructure and cortical anatomy), the following emerged as partial or complete mediators: axial diffusivity within regions of the right anterior (29% of the genetic effect) and right superior corona radiate (21% of the genetic effect); for thickness, a region within the left dorsomedial prefrontal cortex (24% of the genetic effect); for surface area, a region within the right lateral temporal cortex (22% of the genetic effect). Of the 6 cognitive domains, 3 emerged as significant mediators of ADHD PRS → hyperactivity –impulsivity symptoms: working memory (28% of the genetic effect), IQ (20% of the genetic effect) and focused attention (17% of the genetic effect). These mediators fully explained the association between ADHD PRS and hyperactivity–impulsivity symptom. Sustained attention, processing speed, and perseverative/impulsive responding were not significant mediators. In serial mediation analyses (polygenic risk → brain regions → cognition → symptoms), 2 potential pathways emerged. For mediation analyses of neuroimaging data, used permutation and voxelwise *p* < .05. Results mostly held when analyses repeated combining the 2 largest subpopulations; with medication as a covariate, excluding those with comorbid disorders and confining analyses to 1 member of each family. Applied a false discovery rate and indicate the results that survived at q < 0.05.
ASDt, OTHER	36. Serdarevic et al (2020)^64^	Generation R study, The Netherlands N = 1,174–1,921 participants Children were assessed in infancy (9 – 20 wk) and at age 6 y 49% female, 51% male Population sample European ancestry	PRS calculation based on 6 pT (0.01–1)	Neuromotor functioning assessed during in-person home visits using modified Touwen's Neurodevelopmental Examination.^115^ Separate versions used for 9- to 15-wk- old and 16- to 20-wk-old infants Overall scale and Senses, Responses, Hypertone, Hypotone, Tone subscales. Tone included both active and passive muscle strength. Parent-rated autistic traits at age 6 y using the Social Responsiveness Scale Covariates: age, sex, PCs	The ADHD PRS did not predict neuromotor functioning total or subscales after Bonferroni correction; it predicted "Senses and other" subscale nominally (b =0.43, Cl = 0.001 –0.06; *p* =.04, R^2^ = 0.01%). ADHD PRS did not predict autistic traits in whole sample. ADHD PRS predicted autistic traits in boys only (pT < 0.10; b = 0.176, Cl = 0.09–0.27, *p* < .001) after correction for multiple testing, but not in girls. Models that were adjusted for the autism or schizophrenia PRS did not change results. Bonferroni-corrected significance threshold of *p* < .005 applied.
ADHDt, BRAIN, NEUROPSYCH	37. Shen et al (2020)^65^	IMAGEN Study, France, UK, Ireland, Germany N = 1,790 participants Assessed at baseline at age 14 y and at follow-up at 16 y 49% female; 51% male Population sample Ancestry not described	pT <.50	Parent-rated Strengths and Difficulties Questionnaire hyperactivity –inattention subscale^73^ ages 14 and 16 y Neuropsychological variables: working memory errors assessed using Cambridge Neuropsychological Testing Automated Battery^116^ through a self-ordered searching task at age 14 y Delay discounting assessed using the Monetary Choice Questionnaire^117^ which includes items pitting a smaller intermediate reward against a larger delayed reward at age 14 y Intrasubject variability was the SD of reaction time in successful go tasks in the stop signal functional MRI task.^118^ Covariates: age, sex, and site; analyses on GMV also controlled for handedness and total intracranial volume, nbjudifbujd	ADHD PRS was associated with higher ADHD total trait score at age 14 (r = 0.14, df = 1779, *p* < .001, 95% Cl = 0.097, 0.188), working memory errors (r = 0.07, df = 1779, *p* = .002, 95% Cl = 0.026, 0.121) and delay discounting rate (r = 0.06, df = 1779, *p* = .007, 95% Cl = 0.021, 0.109). For lower gray matter volume, the ADHD PRS associated only with the posterior occipital cluster (r = −0.06, df = 1777, *p* = .009, 95% Cl = −0.106, −0.015). Nonsignificant associations not described in publication. Significance threshold not given.
ADHD, ADHDt, BRAIN, NEUROPSYCH	38. Hermosillo et al (2019)^66^	Community recruited children, USA N =196 ADHD participants, 28% female, 72% male N = 119 non-ADHD control participants, 46% female, 54% male Age range = 7 –13 y, mean = 10.38 y (SD = 1.55) Community sample enriched for ADHD European ancestry	PRS calculation based on pT 0.5 (4 other thresholds tested in replications)	ADHD diagnoses were best- estimate research diagnoses from parent semi-structured clinical interviews, clinical observation, and parent/ teacher rating scales. Parent-reported ADHD traits using a latent variable derived from 5 commonly used scales Teacher-reported ADHD traits using a latent variable derived from 3 commonly used scales Working memory assessed using digit span backward, spatial span backward, and IM-back task. MRI-based resting functional connectivity in a targeted set of subcortical structures. In total, 6 circuits involving subcortical regions: left and right caudate, left and right nucleus accumbens, left and right amygdala. Covariates: age, sex, PCs	PRS statistically predicted ADHD diagnosis (b = 0.153, SE = 0.073 SE, *p* = .038) and parent-reported symptoms (b = 0.138, SE = 0.059, *p* = .020) but not teacher-rated symptoms. ADHD PRS did predict working memory (b = 2.194, SE • *p* = .001). ADHD PRS associated significantly with connectivity between the left caudate nucleus and a cluster within the intraparietal sulcus (b = .467, SE = 0.152, *p* = .002), also reported as a significant correlation (r = 0.026, SE = 0.162) and significantly associated with a cluster of regions in the right nucleus accumbens with connectivity to cortex (b = 0.270, SE = 0.117, *p* = 0.021). No significant associations of the ADHD PRS with connectivity of the right caudate nucleus; with connectivity between brain regions and either the left or the right amygdala; or with the connectivity of different clusters correlated with the left nucleus accumbens. A mediation model showed that the PRS–ADHD diagnosis association was suppressed by 60% when the connectivity of a circuit (the connectivity between the left caudate nucleus and the right parietal cortex) was included in the model. Effect sizes were similar for both sexes. No other mediation models showed a significant impact of any of the other connectivity circuits on the ADHD PRS–ADHD diagnosis, ADHD PRS–ADHD symptoms, or ADHD PRS –working memory associations. Results reported as similar when current or previous medication use included in the models, when the sample was sex-matched and with other PRS pT. Permutation testing was applied.
ADHD, ASD	39. LaBianca et al (2020)^68^	Families with multiple individuals with ASD or ADHD recruited through adult psychiatric clinics, Denmark N = 39 multiplex families with 268 individuals, including first-and second-degree relatives of all ages up to 4 generations Age range = 7 –13 y, mean = 10.38 y (SD = 1.55) Northern European ancestry Clinical sample and family relatives	No pT significance threshold	Diagnoses of ASD, ASD, or combined ASD and ADHD, based on *ICD-10* Affected status contingent on PRS score PRS score had Danish samples removed. Covariates: sex, age	ADHD PRS significantly predicted ASD, ADHD, and combined ASD and ADHD. No further information was provided. A significant association was found between the ADHD PRS and being a patient, and affected relatives and unaffected relatives (*p* = .03) using the Kruskal –Wallis ranked sum test.
ADHD	40. Demontis et al (2019)^17^	iPSYCH, a population based case-cohort sample including all singletons born in Denmark between May 1981 and December 2005. European ancestry Psychiatric Genomic Consortium (PGC) includes trio and case control samples. Only European ancestry individuals included in PRS analyses N = 18,298 biologically independent PGC individuals (n = 5,599 cases; n = 12,699 controls) N = 37,076 biologically independent iPSYCH individuals (n = 14,584 cases; n = 22,492 controls)	10 pT were used (from 5 x 10^-8^ – 1). PRS in the iPSYCH sample were achieved with 5 leave-one-out analyses, ie, 4 of 5 groups used as training datasets for estimation of SNP weights while estimating PRS for the excluded target group.	PRS prediction considered the following: within iPSYCh, within PGC, and across all using leave-one-out analysis. iPSYCH cases diagnosed by psychiatrists at in-or out-patient clinics mostly with ICD-10 identified using a Danish Psychiatric Register Controls randomly selected from iPSYCH without ADHD or moderate/severe mental retardation Individuals with a diagnosis of moderate-to-severe mental retardation were excluded from both cases and controls Diagnoses of ADHD derived from range of published instruments in PGC samples Covariates: batch effects, genotyping wave, and PCs	ADHD PRS predicted ADHD across all target samples compared to controls or pseudo-controls. Within iPSYCH (using 5-fold cross-validation), mean of maximum variance explained by ADHD PRS using estimated PRS Nagelkerke's R^2^ was 5.5% (SE = 0.0012), range = 0.047–0.06. Within iPSYCH, OR = 1.56, 95% Cl = 1.53–1.60. Within PGC (with iPSYCH as discovery sample), OR = 1.26 , 95% Cl = 1.22 – 1.31 variance explained on liability scale 0.0103, *p* = 2.4 E^−35^) Across PGC and iPSYCH waves, average variance explained on liability scale = 0.0371 (SE = 0.0029) Increasing deciles of ADHD PRS were associated with increasing OR for ADHD, both for iPSYCH and PGC.
ADDICTION, EA, EXTERNALISING, MH, PHYSICAL	41. Du Rietz *et al.* 2018^68^	UK Biobank, UK N = 135,726, age = 40–73 y; mean = 56.79 y (SD = 7.96) 53% female, 47% male European ancestry Population sample In analyses, controls were individuals without ICD-10 or self-reported diagnosis of alcohol dependency, anxiety disorder, depressive disorder, BD, or schizophrenia and did not take lithium, antidepressants, or antipsychotics	PRS calculation based on multiple pT between 0 and 0.5 at increments of .001	BMI using height and weight General cognitive ability obtained by 2-minute verbal-numerical reasoning test Neuroticism measured with Eysenck Personality Inventory Neuroticism Scale–Revised^119^ Anxiety and depressive disorders, bipolar disorder and schizophrenia identified either through self-report or *ICD-10* codes. Alcohol intake frequency (via self-report question); alcohol-related diagnosis through either self-report or ICD-10 codes. Smoking accessed through hospital records Risk taking coded dichotomously based on yes/no answer to "Would you describe yourself as someone who takes risks?" Covariates: birthplace, age, sex, batch, PCs	ADHD PRS significantly positively predicted BMI (R^2^ = 0.45%; *p* = 4.5 x 10^–129^), cognitive ability (R^2^ = 0.38%; *p* = 4.5 x 10^−36^), alcohol intake frequency (R^2^ = 0.09%; *p* = 8.1 x 10^−29^), alcohol dependency (R^2^ = 0.21%; *p* = 4.5 x 10^–6^), tobacco use (R^2^ = 0.33%; *p* = 4.2 x 10^–21^), risk taking (R^2^ = 0.12%; *p* = 9.3 x 10^−25^), neuroticism (R^2^ = .09%; *p* = 2.2 x 10^−24^), depressive disorder (R^2^ = .11%; *p* = 2.2 x 10^–13^), and height (R^2^ = .03%; *p* = 8.7 x 10−20). ADHD PRS did not significantly predict anxiety disorder, bipolar disorder, or schizophrenia. Within neuroticism, the items were also studied. ADHD PRS significantly predicted mood swings (R^2^ = 0.002%), fed-up feelings (R^2^ = 0.20%), feelings of loneliness and isolation (R^2^ = 0.19%), miserableness (R^2^ = .13%), irritability (R^2^ =0 .09%), being tense/high-strung (R^2^ = 0.07%), guilty feelings (R^2^ = 0.05%), and having easily hurt feelings (R^2^ = 0.05%). It did not predict being a nervous person or a worrier, suffering from nerves, or often worrying after embarrassment. Secondary analyses showed there were no significant sex x PRS interaction effects. Of 8 control phenotypes, included to check for specificity, ADHD PRS significantly and negatively predicted height (R^2^ = 0.03%) and age (R^2^ = 0.03%) but not the other 6 control phenotypes. Significance threshold of *p* < 4.5 x 10–4 was applied.
ADHD, MH	42. Martin *et al.*, 2018^69^	The Child and Adolescent Twin Study in Sweden (CATSS), Sweden. CATSS Registry diagnoses; n = 217 – 443; unaffected n = 13,029–13,247 CATSS screening diagnoses n = 296 – 1,226; unaffected n = 2,083–12,228 Avon Longitudinal Study of Parents and Children (ALSPAC), UK ALSPAC algorithm diagnosed n = 199 – 724; unaffected n = 1,728–2,732 Both population samples Both European ancestry	Primary analyses using pT *p* < 0.1; analyses repeated on 4 other pT	ADHD, any anxiety disorder, any depression disorder or any anxiety or depressive disorder. CATSS had both registry-based ICD-10 clinical diagnoses (captured from ages 9–22 y) and screening-based diagnoses based on parent-/self-rated items from the Autism-Tics, ADHD and Other Comorbidities inventory (ATAC) (assessed at ages 9 or 12 y)^100^ ALSPAC had algorithm-based diagnoses based on a semistructured interview, the Development and Well-Being Assessment (DAWBA^72^ at ages 7, 10, 13 and 15 y from parents. Self ratings were also obtained for anxiety and depression at 15 and 18 y. Covariates: age, PCs	The ADHD PRS consistently predicted ADHD diagnoses using registry clinical diagnoses (OR = 1.39, 95% Cl = 1.26–1.54, *p* = 7.2E-11, screening research diagnoses (OR = 1.25, 95% Cl = 1.17–1.34, *p* = 2.8E-11) and algorithm-based research diagnoses (OR = 1.76, 95% Cl = 1.51–2.05, *p* = 4.9E-13). The ADHD PRS predicted anxiety disorders using registry clinical diagnoses (OR = 1.16, 95% Cl = 1.02–1.32, *p* = .020) and algorithm-based research diagnoses (OR = 1.20, 95% Cl = 1.08–1.33, *p* = .00046) but not screening research diagnoses. The ADHD PRS predicted depressive disorders only using algorithm-based research diagnoses (OR = 1.19, 95% Cl = 1.06–1.33, *p* = .0027) and not using registry clinical or screening research diagnoses. The ADHD PRS consistently predicted any anxiety or depressive disorder using registry clinical diagnoses (OR = 1.16, 95% Cl = 1.04–1.29, *p* = .0062), screening research diagnoses (OR = 1.12, 95% Cl = 1.01–1.25, *p* = .031), and algorithm-based research diagnoses (OR = 1.17, 95% Cl = 1.07–1.27, *p* = .00063). Repeated analyses using other pT showed similar results. A significance threshold of *p* < .05 was applied.
MH	43. Rice *et al.* 2019^70^	The Avon Longitudinal Study of Parents and Children (ALSPAC), UK N = 5416 adolescents with PRS scores and depression data on more than 1 assessment point between 10 and 18 y 47% male; 53% female Population sample European ancestry	pT < 0.50	Self-report depressive symptoms using the short Mood and Feelings Questionnaire,^120^ 6 ages (10.5, 12.5, 13.5, 16.5, 17.5, and 18.5 y) Categorized individuals scoring above/below clinical cut-off of scale Family history measured as the number of family members with a history of depression or schizophrenia weighted by relatedness (first or second-degree relative) Three trajectory classes identified: persistently low (73.7%), later-adolescence onset (17.3%), and early-adolescence onset (9.0%).	The AHDH PRS did not correlate significantly with family history for major depression or schizophrenia (both *p* > .05). ADHD PRS predicted the early-adolescence–onset depression class (OR, 1.32, 95% Cl = 1.13–1.54, *p* < .001) In multi-PRS analyses including also the schizophrenia and MDD PRS, the ADHD PRS still predicted the early (OR = 1.27, 95% Cl = 1.08–1.50, *p* = .003) ADHD PRS did not predict the later-onset depression trajectory class in either the univariate analysis or the multi-PRS analysis. Analyses that were re-run including PCS, adjusting for missing phenotypic data, and adjusting for missing genetic data, showed similar findings. Significance threshold of *p* < .05 applied.
ADHDt, EA, EXTERNALIZING, OTHER, SES	44. Zwicker *et al.* 2020^71^	Families Overcoming Risks and Building Opportunities for Well-being (FORBOW study, Canada N= 297 participants aged 5 – 27 y (mean = 13.5, SD = 4.4) 53% female; 47% male Sample enriched for offspring of parents with depression, bipolar disorder, and schizophrenia 90% European ancestry; 10% non-European ancestry	pT<.50. Analyses repeated using other pT	Total adversity score calculated as mean of 10 binary indicators: biological mother's education, biological father's education, home ownership status, annual household income, emotional abuse, physical abuse, sexual abuse, neglect, exposure to violence at home, bullying. Socioeconomic and victimization adversity subscales also studied ADHD symptoms: <18 y: Kiddie Schedule for Affective Disorders and Schizophrenia (KSADS)– Present and Lifetime Version; >18 y: Structured Clinical Interview for DSM-5 Externalizing symptoms score from KSADS interview IQ assessed with Wechsler Abbreviated Scale of Intelligence–Second Edition^121^ or Wechsler Preschool and Primary Scale of Intelligence Covariates: age, sex, time in the study, PCs	ADHD PRS was associated with ADHD symptoms (β = 0.21, 95% Cl = 0.10–0.32, *p* < .001, R^2^ = 3.0%) and externalizing behaviors (β = 0.23, 95% Cl = 0.12–0.34, *p* < .0001; R^2^ = 4.0%; r = 0.22, *p* < .05). ADHD PRS was associated with adversity (b = 0.23, 95% Cl = 0.13–0.34, *p* < .0001; R^2^ = 4.0%) as well as the socioeconomic adversity (b = 0.10, 95% Cl 0.01 –0.20, *p* = .028; R^2^ = 2.0%) and victimization adversity subscales (b = 0.24, 95% Cl = 0.12–0.35, *p* < .0001 R^2^ = 3.3%). ADHD PRS did not significantly associate with IQ or with family history for schizophrenia. Mediation models to test the ADHD PRS → adversity association showed that externalizing symptoms mediated 22% of the total effect of ADHD PGS on adversity. IQ did not mediate the ADHD PRS → adversity association. Associations held when run separately in individuals with and without ADHD; on the subset of participants under age 17 y; after excluding offspring of control parents; among the subset of participants with a biological parent with mental illness; and on the subset with self-reported European descent. Univariate PRS analysesused *p* < .003 (Bonferroni significance threshold corrected for multiple tests).

**Note**: Sample N are given for genotyped polygenic risk score (PRS) sample used in analyses. Principal components (PCs) to control for population stratification. pT, single nucleotide polymorphism p value threshold for PRS: if authors did not select a primary pT, results are reported for most significant pT. Outcome categories were labeled as: Attention-Deficit/Hyperactivity Disorder diagnosis (ADHD); ADHD traits (ADHDt); Substance and non–substance-based addiction phenotypes (Addiction); Autism diagnosis (ASD); Autistic traits (ASDt); Imaging-based assessments of brain variables including structure, function and connectivity (Brain); Educational attainment phenotypes (EA); Externalizing behaviors (Externalizing); Mental health phenotypes (MH); Neuropsychological phenotypes (Neuropsy); Uncategorized phenotypes (Other); Physical health phenotypes (Physical); and Socioeconomic status variables (SES) AHPVT = Add Health Picture Vocabulary Test; BFIS = Bullying and Friendship Interview Schedule; BRIEF = Behavior Rating Inventory of Executive Function; CBCL = Child Behavior CheckList/6–18; CES-D = Center for Epidemiologic Studies Depression scale; DAWBA = Development And Well-Being Assessment; EDI-2 = Eating Disorder Inventory–2; GWAS = genome-wide association study; ICD [ International Statistical Classification of Diseases and Related Health Problems; MRI = magnetic resonance imaging; PC = principal component; PCA = principal component analysis; PRS = polygenic risk score; pT = p value threshold of discovery GWAS as used for ADHD PRS; SCDC = Social and Communication Disorders Checklist; SDQ = Strengths and Difficulties Questionnaire; SWAN = Strengths and Weaknesses of ADHD Symptoms and Normal behavior rating scale; WURS = Wender Utah Rating Scale.

**TABLE 2:** Domains (1_5) and Criteria List for the Quality Assessment of Included Studies

Criteria
**1. Study participation; Study sample adequately represents the population of interest**
**(A)** Description of the key characteristics of the study population (distribution by age, gender, and ancestry/ethnicity)
**(B)** The sampling frame and recruitment are described, including characteristics of the place of recruitment or authors clearly reference where this information can be found
**(C)** Inclusion and exclusion criteria are described or authors clearly reference where this information can be found
**(D)** Information about participation at baseline and potential attrition (for genetic data) are described, or authors clearly reference where this information can be found
**2. Predictor measurement; ADHD PRS is adequately measured**
**(E)** Description of genetic data collection (eg, blood, saliva) and genotyping (array) is provided, and target sample was not part of ADHD GWAS
**(F)** Genetic data were subject to adequate quality control (minor allele frequency, missing rate, relatedness participants, sex mismatch, and genotype quality), an up-to-date imputation method and an established reference panel was used
**(g)** The ADHD PRS is adequately calculated (eg, pruning/clumping of SNPs)
**3. Outcome measurement; outcome of interest is measured in a similar way for all participants**
**(H)** A clear definition of the outcome measures is provided
**(I)** Several indications are provided for the validity and reliability of the outcome measure, or a reference is provided.
**(J)** The method and setting of outcome measurement is the same for all study participants
**4. Confounding measurement; important potential confounders are appropriately accounted for**
**(K)** Age, gender, and socioeconomic status are accounted for in the analysis
**(L)** Population stratification and potential batch effects are accounted for in the analysis
**(M)** In the case of clinical samples, treatment and comorbidity are accounted for in the analyses
**5. Analysis and data presentation; statistical analysis is appropriate**
**(N)** There is sufficient presentation of the data to assess the adequacy of the analytic strategy
**(O)** The number of participants in the target sample supports sufficient statistical power (N > 400)
**(P)** The selected statistical model is adequate for the design of the study
**(Q)** There is no evidence of selective reporting of results, and proper correction for multiple testing was applied

**Note**: ADHD = attention-deficit/hyperactivity disorder; PRS = polygenic risk score; SNP = single-nucleotide polymorphism.

**TABLE 3:** Definitions of Levels of Evidence

Level of evidence	
Strong	Consistent findings (≥75%) in at least 2 high-quality studies
Moderate	Consistent findings (≥75%) in 1 high-quality study and at least 1 study of lower quality
Weak	Findings in 1 high quality study or consistent findings (≥75%) in at least 3 studies of lower quality
Inconclusive	Inconsistent findings irrespective of study quality, or less than 3 lower-quality studies available

**Note**: “(≥75%)”: Within a category, at least 75% of the findings of studies had to agree on the existence and direction of the relation between the attention-deficit/hyperactivity disorder (ADHD) polygenic risk score (PRS) and the outcome measure.

**TABLE 4:** Quality Assessment Results of Included Studies

Domains	1) Study participation				2) ADHD PRS			3) Outcomes			4) Confounders			5) Analysis, data presentation				N
						
Criteria	A	B	C	D	E	F	G	H	I	J	K	L	M	N	O	P	Q	bias
Stojanovski *et al*. 2019^30^	+	+	+	+	+	+	+	+	−	+	+	+	+	+	−	+	+	0
Albaugh *et al*. 2019^29^	+	+	+	+	−/+	+	+	+	−/+	+	+	+	NA	+	+	+	+	0
Burton *et al*. 2019^33^	+	+	+	+	+	+	+	+	+	+	−/+	+	−	+	+	+	+	0
Jansen *et al*. 2019^31^	+	+	−/+	+	+	+	+	+	+	+	−/+	+	−/+	+	+	+	+	0
Li 2019a^32^	+	+	+	+	+	+	+	+	−	+	+	+	NA	+	+	+	+	0
Gialluisi *et al*. 2019^34^	+	−	+	−	+	+	−/+	+	−	−	−	−/+	−	+	+	+	+	2
Rietveld and Patel 2019^35^	+	−	−/+	−	−	−	−	+	−/+	−/+	+	+	NA	+	+	+	+	2
Piasecki *et al*. 2019^36^	+	+	+	+	+	+	+	+	−	+	−/+	+	NA	+	+	+	−/+	0
Torske *et al*. 2019^37^	+	+	+	+	+	+	+	+	−/+	+	−/+	+	−	+	−	−/+	−	1
Nigg *et al*. 2019^38^	+	+	+	+	+	+	+	+	−	+	−/+	+	+	+	−/+	+	−/+	0
Dickinson *et al*. 2019^39^	+	+	+	−	+	+	+	+	+	+	−/+	+	−	+	−/+	+	−/+	0
Cabana–Domínguez *et al*. 2019^40^	+	+	+	+	+	+	+	+	+	−/+	−	+	−	−/+	+	+	−/+	0
Ohi *et al*. 2020^41^	+	+	+	+	+	+	+	+	+	+	−	+	−	+	−	+	−/+	0
Mooney *et al*. 2020a^42^	+	−/+	+	+	+	−/+	+	+	+	+	−/+	+	−/+	+	−	+	+	0
Vuijk *et al*. 2019^43^	+	−/+	+	+	+	+	+	+	+	+	−/+	+	+	+	−	+	+	0
Li 2019b^44^	+	+	+	+	+	+	+	+	+	+	+	+	NA	+	+	+	−/+	0
Riglin *et al*. 2019^45^	+	+	+	+	+	+	+	+	+	+	+	−	NA	+	+	+	−/+	0
Grigoroiu–Serbanescu *et al*. 2019^46^	+	−/+	−/+	−	−/+	−/+	+	+	+	−/+	−	−	−	−/+	+	−/+	+	1
Wimberley *et al*. 2019^47^	+	+	+	+	+	+	+	+	+	+	+	+	−/+	+	+	+	+	0
Riglin *et al*. 2020^48^	+	+	−/+	+	+	+	+	+	+	+	−	−	NA	+	+	+	−/+	1
Barker *et al*. 2019^49^	+	+	−/+	+	−/+	+	+	+	+	+	−/+	+	NA	+	+	+	+	0
De Zeeuw *et al*. 2019^50^	−/+	+	+	+	+	+	+	+	+	+	−/+	+	NA	+	+	+	+	0
Yao *et al*. 2019^51^	+	+	+	+	+	+	+	+	+	+	−/+	+	−	+	+	+	−/+	0
Rabinowitz *et al*. 2018^52^	+	+	−/+	+	+	+	+	+	+	−/+	−/+	+	−	+	+	+	+	0
Taylor *et al*. 2019^53^	+	+	+	+	+	+	+	+	+	+	−/+	+	NA	+	+	+	+	0
Alemany *et al*. 2019^54^	+	+	+	+	+	+	+	+	+	+	−/+	+	NA	+	+	+	+	0
Gurriarán *et al*. 2018^55^	+	+	+	+	+	+	+	+	+	−/+	−/+	+	−	+	+	+	+	0
Szekely *et al*. 2018^56^	+	−/+	−/+	+	+	+	+	+	+	−	−/+	+	NA	−/+	+	+	+	0
Nigg *et al*. 2018^57^	+	+	+	+	+	+	+	+	+	+	−/+	−/+	+	+	+	+	+	0
Hawi *et al*. 2018^58^	+	−	+	+	+	+	+	+	+	+	−/+	+	−	+	−	+	+	0
Taylor *et al*. 2018^59^	+	+	+	+	+	+	+	+	−	+	−/+	+	NA	+	+	+	−/+	0
Selzam *et al*. 2019^60^	+	+	+	+	+	+	+	+	−/+	+	+	+	NA	+	+	+	+	0
Schoeler *et al*. 2019^61^	+	+	+	+	+	+	+	+	−	+	−/+	+	NA	+	+	+	+	0
Mooney *et al*. 2020b^62^	+	−/+	+	−/+	+	+	+	+	+	+	−/+	+	−/+	+	−/+	+	+	0
Sudre *et al*. 2018^63^	−	−	+	−	+	−/+	+	+	+	+	−/+	−/+	+	+	−/+	+	+	1
Hermosillo *et al*. 2020^33^	+	+	−/+	+	+	+	+	+	+	+	−/+	+	−/+	+	−/+	+	+	0
LaBianca *et al*. 2020^67^	−	+	−	+	−	−	+	+	+	+	−/+	−	−	−/+	−/+	+	+	2
Serdarevic *et al*. 2020^64^	−/+	+	+	+	+	+	+	+	+	+	+	+	NA	+	+	+	+	0
Shen *et al*. 2020^65^	+	+	+	+	−/+	+	+	+	−/+	−	−/+	−	−/+	+	+	+	−/+	1
Demontis *et al*. 2019^17^	+	+	−/+	−/+	+	+	+	+	+	−	−	+	−	+	+	+	+	0
Du Rietz *et al*. 2018^68^	+	+	+	+	+	+	+	+	−/+	+	−/+	+	NA	+	+	+	+	0
Martin *et al*. 2018^69^	+	+	+	+	+	+	+	+	+	−	−/+	+	NA	+	+	+	−/+	0
Rice *et al*. 2019^70^	+	+	+	+	+	+	+	+	+	+	−/+	+	NA	+	+	+	−/+	0
Zwicker *et al*. 2019^71^	+	+	+	+	+	+	+	+	−/+	+	−/+	+	NA	+	−/+	+	+	0
