# Supplementary figures and images for: Systematic Review: How the Attention-Deficit/Hyperactivity Disorder Polygenic Risk Score Adds to Our Understanding of ADHD and Associated Traits
Source: J Am Acad Child Adolesc Psychiatry. Author manuscript; Available in PMC 2024 Jun 10. (PMC11164195; doi:10.1016/j.jaac.2021.01.019)

Figure S1. Bar chart summarising number of studies per category, and strength of association.

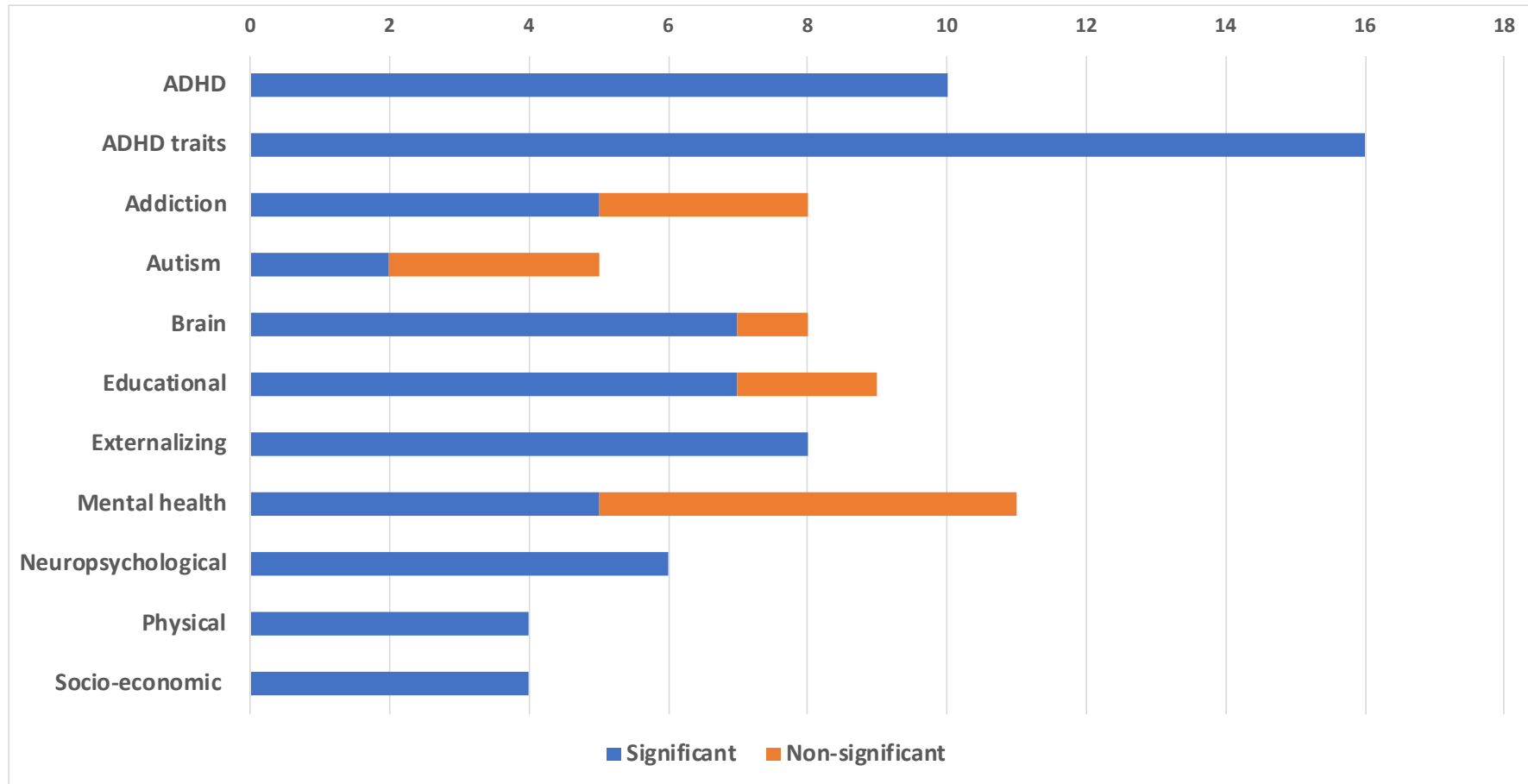

Supplement: Supp Mat 1 [file NIHMS1978791-supplement-Supp_Mat_1.pdf]
