# Supplementary material for: Systematic Review: How the Attention-Deficit/Hyperactivity Disorder Polygenic Risk Score Adds to Our Understanding of ADHD and Associated Traits
Source: J Am Acad Child Adolesc Psychiatry. Author manuscript; Available in PMC 2024 Jun 10. (PMC11164195; doi:10.1016/j.jaac.2021.01.019)
Supplement: Supp Mat 2 [file NIHMS1978791-supplement-Supp_Mat_2.pdf]

Table S1: Measured traits for each category

Category (N studies)	Measured traits (study number in Table 1)
ADHD diagnosis (10)	• Diagnostic and Statistical Manual of Mental Disorders fourth edition (DSM-IV) (3[#], 18[#], 29[#], 30[#])<br>• Retrospectively self-reported ADHD symptoms keyed to the DSM-IV (4)<br>• ICD-10 (15[#], 39[#], 40[#], 42)<br>• Retrospectively Wender Utah Rating Scale (WURS), Kiddie-SADS clinical interview. Assessment of childhood ADHD was made by clinicians (18[#])<br>• Conners' Parent Rating Scale (30[#])<br>• Best estimate research diagnoses from parent semi-structured clinical interviews, clinical observation and parent/teacher rating scales (38[#])<br>• Autism-Tics, ADHD and Other Comorbidities inventory (ATAC) (42)<br>• Development and Well-Being Assessment (DAWBA) (42)
ADHD traits (16)	• Composite score of the Development and Well-Being Assessment (DAWBA) and the Strengths and Difficulties Questionnaire (SDQ) (1)<br>• Diagnostic and Statistical Manual of Mental Disorders fifth edition (DSM5) (2)<br>• Strengths and Weaknesses of ADHD Symptoms and Normal Behavior Rating Scale (SWAN) score: Total, inattentive and hyperactive/impulsive subscales (5)<br>• Dimensional score on an ADHD latent variable captured from hyperactivity and inattention subscales of four published ADHD scales (10[#])<br>• DSM-IV Axis 1 diagnoses; a range of parent-rated dimensional published scales of psychopathology (15[#])<br>• DSM-IV items retrospectively (16)<br>• Wender Utah Rating Scale (WURS) and items from the Kiddie-SADS clinical interview (18[#])<br>• Child Behavior Checklist (CBCL) Attention Problem scales (22, 26)<br>• Teacher Report Form (TRF) Attention Problem scales (22)<br>• The Autism-Tics, AD/HD and Other Comorbidities Inventory (A-TAC) (25)<br>• Separate parent and teacher-rated ADHD symptom latent variables derived from data on 3-4 published ADHD measures that capture inattention and hyperactivity (29[#])<br>• SDQ (32)

	• Conners’ Parent Rating Scale (32)<br>• Inattention and hyperactivity disorder symptoms by clinician administered Diagnostic Interview for Children and Adolescents for parents (35[#])<br>• Strengths and Difficulties Questionnaire (SDQ) (37)<br>• Parent-reported ADHD traits using a latent variable derived from five commonly used scales (38[#])<br>• Teacher-reported ADHD traits using a latent variable derived from three commonly used scales (38[#])<br>• ADHD symptoms: under 18 yrs: Kiddie Schedule for Affective Disorders and Schizophrenia (KSADS)– Present and Lifetime Version; over 18 yrs: Structured Clinical Interview for DSM-5 (44)
Addiction (8)	• Lifetime DSM-IV criteria for alcohol abuse or dependence were assessed as the presence of at least 1 of the 4 items pertaining to alcohol abuse, and/or 3 of the 7 items pertaining to alcohol dependence occurring together in 12-month period (4)<br>• Gambling: answering yes or no to “Have you ever bought lottery tickets, played video games or slot machines for money, bet on horses or sporting events, or taken part in any other kinds of gambling for money?”; and (if yes to the previous question), answer of yes or not to: “Has your gambling ever caused serious financial problems or problems in your relationships with any of your family members or friends?” (8)<br>• Cocaine dependence DSM-IV (12[#])<br>• Presence of substance use disorder history (15[#])<br>• Addiction categorized first by alcohol, cannabis, and other illicit drugs and second categorized into severity into use, abuse and addiction (nicotine use not included) (19)<br>• Composite International Diagnostic Interview-University of Michigan Version (CIDI-UM), National Survey on Drug Use and Health (NSDUH) (24)<br>• Substance use disorder DSM-IV (27[#])<br>• Alcohol addiction ICD-10 (41)<br>• Smoking through hospital records (41)
Autism/autistic traits (5)	• DSM-IV ASD diagnosis (3[#])

	• Social Responsiveness Scale (9[#], 15[#], 36)<br>• ICD-19 (39[#])
Brain measures (8)	• Neuroanatomic imaging, and imaging of white matter tract microstructure (1)<br>• Total brain volume (TBV) and subcortical structures (14[#])<br>• Voxel-based morphometry measures of whole-brain grey matter (21)<br>• Neural responses to reward anticipation and reward outcome from activation maps from a Monetary Incentive Delay fMRI task (21)<br>• Total brain volume (TBV), cortical gray matter (GM), total white matter, subcortical GM, ventricular volume, cerebellum, amygdalahippocampus complex, caudate, putamen and thalamus (26)<br>• Longitudinal growth in volume across 2 time points modeled linearly for 4 brain divisions: cerebral cortex, basal ganglia, cerebellum, cerebral white matter, and one region of interest: the right lateral prefrontal cortex (28[#])<br>• Neuroanatomic imaging, and imaging of white matter tract microstructure (35)<br>• Stop signal functional MRI task (37)<br>• MRI-based resting functional connectivity in left and right caudate, left and right nucleus accumbens, left and right amygdala (38[#])
Educational attainment (9)	• Cognitive ability, measured by Add Health Picture Vocabulary Test (AHPVT) (4)<br>• Educational attainment, measured by the question 'what is the highest level of education that you have achieved to date? (4, 22)<br>• Eight outcomes relating to word reading, spelling, rapid naming, and phonology that are considered core deficits in dyslexia: Word reading (WRead), nonword reading (NWRead), and word spelling (WSpell), Phoneme awareness (PA), digit span (DigSpan, a measure of verbal short-term memory), and rapid automatized naming of letters (RANlet), digits (RANdig), and pictures (RANpic) (6)<br>• Wide-Range Achievement Test [WRAT] reading subtest and Wechsler Adult Intelligence Scale [WAIS] used for cognitive assessments (11[#])<br>• Wechsler Intelligence Scale for Children–Fourth Edition and the Wechsler Adult Intelligence Scale–4th Edition (15[#], 35[#])

	• Word Reading and Numerical Operations of the Wechsler Individual Achievement Test–Third Edition (WIAT III) (15[#])<br>• Whether education was completed by age 23 years or not (15[#])<br>• Cito score, a Dutch nationwide standardized educational achievement test (22)<br>• Wechsler Intelligence Scale III, verbal and nonverbal ability (32)<br>• UK General Certificate of Secondary Education; GCSE (32)<br>• General cognitive ability obtained by 2-minute verbal-numerical reasoning test (41)<br>• IQ assessed with Wechsler Abbreviated Scale of Intelligence – Second Edition or Wechsler Preschool and Primary Scale of Intelligence (44)
Externalizing behaviors (8)	• Irritability captured with latent variable based on two subscale scores: anger and modified soothability from the Temperament in Middle Childhood Questionnaire (TMCQ, and an oppositional defiant disorder irritable total score. Latent variables were also created for surgency-approach and sadness-anxiety (10[#])<br>• DSM-IV Axis 1 diagnoses; a range of parent-rated dimensional published scales of psychopathology (15[#])<br>• Aggressive behaviors, non-aggressive rule breaking and substance use behaviors assessed by in-person interviews (16)<br>• Parent-reported data on Development and Well-Being Assessment (DAWBA)[1]—a structured research diagnostic interview—at ages 7, 10, 13 and 15 years (17)<br>• Comorbid oppositional defiant disorder/conduct disorder (ODD/CD) (19[#])<br>• Impulsivity symptoms at age 19 assessed using self-reported Barratt Impulsivity Scale (BIS) (21)<br>• Risk taking coded dichotomously based on yes/no answer to “Would you describe yourself as someone who takes risks?” (41)<br>• Externalizing symptoms score from KSADS interview (44)
Mental health (11)	• Diagnoses based on the DSM-IV, the Center for Epidemiologic Studies Depression (CES-D) Scale, and an abbreviated 4-item version of the Cohen’s Perceived Stress Scale (4)<br>• Whether participant was ‘ever arrested’ (4)<br>• Diagnoses based on the DSM-IV (11[#], 15[#], 18[#])

	• Diagnoses based on DSM5 (13[#])<br>• P-factor based on DAWBA, the Social and Communication Disorders Checklist (SCDC) (20)<br>• 3 subscales (Drive for Thinness, Bulimia, and Body Dissatisfaction) from the Eating Disorder Inventory-2 (EDI-2) (23)<br>• Specific Psychotic Experiences Questionnaire (32)<br>• Neuroticism assessed by Big Five questionnaire (32)<br>• Eysenck Personality Inventory Neuroticism Scale–Revised (41)<br>• Diagnoses based on ICD-10 codes (41, 42)<br>• Development and Well-Being Assessment (DAWBA) (42)<br>• Mood and Feelings Questionnaire (43)<br>• Family history measured as the number of family members with a history of depression or schizophrenia weighted by relatedness (first or second-degree relative) (43)
Neuropsychological constructs (6)	• Behavior Rating Inventory of Executive Function (BRIEF), a 86-item questionnaire. The Behavior Regulation Index (which incorporates 3 subscales: inhibit, shift, and emotional control) and the Metacognition Index (which incorporates 5 subscales: initiate, working memory, plan/organize, organization of materials, and monitor). The Global Executive Composite Index comprised all 8 above subscales (9[#])<br>• Working memory index from the Wechsler Intelligence Scale for Children–Fourth Edition (15[#])<br>• Laboratory measures of working memory, response inhibition, executive functioning, arousal/attention, temporal, information processing, and processing speed (29[#])<br>• Working memory spans assessed through number of correctly recalled digits/tapping patterns (35[#])<br>• Processing speed assessed using visual matching task (from the Woodcock Johnson III Test of Cognitive Abilities) (35[#])<br>• Conners' Continuous Performance Test (35[#])<br>• Cambridge Neuropsychological Testing Automated Battery (37)<br>• Monetary Choice Questionnaire (37)<br>• Working memory assessed using digit span backward, spatial span backward, and N-back task (38[#])

Physical health (4)	• Body mass index (BMI) (4, 21, 32, 41)<br>• Patient-reported hypertension or high blood cholesterol as assessed by a doctor (4)<br>• Height (32)<br>• Self-rated health (RAND Short-Form Health Survey) (32)
Socio-economic variables (4)	• Six later-life US labor market outcomes: currently working for pay, individual earnings (gross individual income), total household wealth (net value of total wealth, excluding second home, if applicable), receiving governmental assistance in the form of social security disability insurance, receiving unemployment or workers' compensation, receiving other governmental transfers (7)<br>• paternal income, maternal education (19[#])<br>• Socio Economic Status: based on maternal age at birth of the first child, maternal and paternal highest education level, and maternal and paternal occupation (32)<br>• Socio-economic adversity scale (biological mother's education, biological father's education, homeownership status, annual household income) (44[#])
Other (9)	• Mild traumatic brain injury (2)<br>• Age of onset BP (18[#])<br>• Parental Substance Use Disorder, parental mental disorder (19[#])<br>• The Structured Interview of Parent Management Skills and Practices Youth-Version (SIPMSP) (24)<br>• The community disadvantage score was calculated using census-tract level items from the 1990 and 2000 Decennial census (24)<br>• Study participation defined as responding to a questionnaire or attending a clinic for which the whole cohort was eligible to participate (31)<br>• Bullying and Friendship Interview Schedule (BFIS) (33)<br>• 568,281 probes assessed for DNA methylation on the MethylationEPIC BeadChip (34[#])<br>• Neuromotor functioning: Touwen's Neurodevelopmental Examination (36)<br>• Victimization adversity scale (emotional abuse, physical abuse, sexual abuse, neglect, exposure to violence at home, bullying (44[#])

Note: [#]clinical sample, or enriched sample
